# Serine Protease Inhibitors in Ticks: An Overview of Their Role in Tick Biology and Tick-Borne Pathogen Transmission

**DOI:** 10.3389/fcimb.2017.00199

**Published:** 2017-05-22

**Authors:** Adrien A. Blisnick, Thierry Foulon, Sarah I. Bonnet

**Affiliations:** ^1^UMR BIPAR INRA-ENVA-ANSESMaisons-Alfort, France; ^2^Centre National de la Recherche Scientifique, Institut de Biologie Paris-Seine, Biogenèse des Signaux Peptidiques, Sorbonne Universités, UPMC Univ. Paris 06Paris, France

**Keywords:** ticks, tick serine protease inhibitors, tick-borne pathogens, tick–host interactions, immune responses

## Abstract

New tick and tick-borne pathogen control approaches that are both environmentally sustainable and which provide broad protection are urgently needed. Their development, however, will rely on a greater understanding of tick biology, tick-pathogen, and tick-host interactions. The recent advances in new generation technologies to study genomes, transcriptomes, and proteomes has resulted in a plethora of tick biomacromolecular studies. Among these, many enzyme inhibitors have been described, notably serine protease inhibitors (SPIs), whose importance in various tick biological processes is only just beginning to be fully appreciated. Among the multiple active substances secreted during tick feeding, SPIs have been shown to be directly involved in regulation of inflammation, blood clotting, wound healing, vasoconstriction and the modulation of host defense mechanisms. In light of these activities, several SPIs were examined and were experimentally confirmed to facilitate tick pathogen transmission. In addition, to prevent coagulation of the ingested blood meal within the tick alimentary canal, SPIs are also involved in blood digestion and nutrient extraction from the meal. The presence of SPIs in tick hemocytes and their involvement in tick innate immune defenses have also been demonstrated, as well as their implication in hemolymph coagulation and egg development. Considering the involvement of SPIs in multiple crucial aspects of tick-host-pathogen interactions, as well as in various aspects of the tick parasitic lifestyle, these molecules represent highly suitable and attractive targets for the development of effective tick control strategies. Here we review the current knowledge regarding this class of inhibitors in tick biology and tick-borne pathogen transmission, and their potential as targets for future tick control trials.

## Introduction

Ticks are among the most common and important vectors of both human and animal pathogens worldwide including some parasites, bacteria and viruses (Dantas-Torres et al., 2012). These obligate hematophagous arthropods are divided into two main families; soft and hard ticks. Tick developmental stages include larval, nymphal and adult forms, all of which—for most species—require blood meals to complete development and enable reproduction. Compared to other hematophagous arthropods, hard –or *Ixodidae-* tick feeding is a slow and complex process, taking several days until repletion, and thus necessitates extended control over the vertebrate host's immune response. Whereas the soft—or *Argasidae*—ticks usually complete a blood meal in less than 1 h (Sonenshine and Anderson, 2014). During this feeding process, all ticks inject saliva and absorb blood alternately. Blood cells are lysed in the midgut lumen and, in contrast to other hematophagous arthropods, further digestion of proteins and other blood molecules occurs intracellularly, taking place within midgut epithelial cells.

Current tick control strategies essentially rely on the use of chemical acaricides and repellents. Their widespread deployment however, has a profound environmental impact (Rajput et al., 2006; De Meneghi et al., 2016), and has led to the emergence of resistance in multiple tick species (Rosario-Cruz et al., 2009; Adakal et al., 2013). New environmentally sustainable approaches providing broader protection against current and future tick-borne pathogens (TBP) are thus urgently needed. To investigate new candidate pathways to fight the spread of these pathogens, a complete understanding of tick biology, tick-pathogen and tick-host interactions is essential. Since the beginning of the twenty first century the continual development of cutting-edge high-throughput methods has enabled the study of genomes, transcriptomes, and proteomes, thus facilitating many diverse biomolecular studies (Metzker, 2010). These tools have been essential in the discovery of specific tick biological gene products. The most studied tick species have been those that present significant human and/or livestock disease risk in the northern hemisphere: *Ixodes scapularis* in the USA (deer; black-legged tick); *Ixodes persulcatus* in Asia and Eastern Europe (Taiga tick); and *Ixodes ricinus* in western and central Europe (sheep tick). Additionally, the *Rhipicephalus (Boophilus) microplus* cattle tick that causes massive damage in Australia, Africa, Central America, and Asia has also been intensively studied.

Most studies have investigated specific tick organ transcriptomes under a variety of conditions, especially tick salivary glands (SGs) (Santos et al., 2004; Francischetti et al., 2005b; Ribeiro et al., 2006; Garcia et al., 2014; Liu et al., 2014) or midgut (Anderson et al., 2008; Chmelar et al., 2016), occasionally eggs or ovaries (Santos et al., 2004), and less frequently hemocytes (Santos et al., 2004; Kotsyfakis et al., 2015), body fat or synganglia (Bissinger et al., 2011; Egekwu et al., 2014). Several comprehensive protein catalogs describing protein diversity in various tick fluids such as saliva (Madden et al., 2004; Cotté et al., 2014; Radulovic et al., 2014; Tirloni et al., 2014a, 2015) or hemolymph (Gudderra et al., 2002; Stopforth et al., 2010), as well as in midgut during feeding (Schwarz et al., 2014; Oleaga et al., 2015), have been compiled, vital to understanding mechanisms implicated in different biological processes such as tick feeding or tick immunity.

Several studies also reported that TBP can influence gene and protein expression in tick, highlighting evidence of molecular interaction between pathogens and the vector (review in Liu and Bonnet, 2014). These studies focused on specific organs including SGs, midgut, ovaries, or on the whole tick during infections with several different pathogens, and reported differential expression of tick's genes links to pathogen transmission. TBP are imbibed by tick when feeding on a pathogen-infected vertebrate host and, once ingested, they directly or not -depending of the pathogen- escape the midgut and invade the SGs and the ovaries for vertically transmitted pathogens (see Liu and Bonnet, 2014). Then, for most TBP, transmission to a new host occurs via the saliva during blood feeding. During both their transmission and development into the vector, TBP undergo developmental transitions and migrations and suffer population losses, to which tick factors surely contribute. In addition, during the prolonged tick-host attachment period, many proteins injected into the host via tick saliva dampen host defenses, thereby creating a favorable environment for survival and propagation of TBP (Brossard and Wikel, 2004; Nuttall and Labuda, 2004; Ramamoorthi et al., 2005; Wikel, 2013).

Many enzyme activity inhibitors were described among the transcripts or proteins detected in these studies, including multiple protease inhibitors often belonging to serine protease inhibitor families. These inhibitors can vary in molecular weight from less than 10 kDa to almost 100 kDa, and can reversibly or irreversibly inhibit their targets via family-specific domains. Their global tissue expression suggests involvement in various important tick biological pathways, including innate immunity, hemolymph clotting formation, blood uptake, digestion, as well as oviposition and egg laying. In addition, tick serine protease inhibitors (tSPIs) also modulate vertebrate host responses during biting, act on hemostasis, immune responses, or angiogenesis. Their implications in these various processes suggest that tSPIs can indirectly influence tick pathogen transmission, and indeed some have been directly experimentally linked with TBP transmission. The aim of the present review is to summarize current knowledge concerning these tSPIs (detailed in Table 1), in order to highlight their role in tick biology, TBP transmission, and to identify putative targets which could contribute to effective tick and TBP control strategies.

**Table 1:** **Tick serine protease inhibitors implicated in both tick biology/physiology and modulation of vertebrate host responses to tick bite, classified according to their inhibitor group (Serpin, Macroglobuline, Kunitz, Kazal), and the corresponding tick species**.

**Inhibitor name**	**Molecular weight (kDa)**	**Inhibitor type**	**Tick species**	**Action**	**References**
**TICK IMMUNE SYSTEM FACTORS**					
TAM	420	α2M	*O. moubata*	Tick immune defense	Kopacek et al., 2000
IrAM	440	α2M	*I. ricinus*	Antimicrobial activity	Buresova et al., 2009
BmCI	6.5	Kunitz	*R. (B.) microplus*	Antimicrobial activity	Lima et al., 2010
DvKPI	62	Kunitz	*D. variabilis*	Antimicrobial activity	Ceraul et al., 2008
Ixodidin	7.1	Trypsin Inhibitor Like (TIL)	*R. (B.) microplus*	Antimicrobial activity	Fogaça et al., 2006
BmSI 6-7	7.4, 7.3	Trypsin Inhibitor Like (TIL)	*R. (B.) microplus*	Antimicrobial activity and tissue preservation	Sasaki et al., 2008
BmTI-A	13.5	Kunitz	*R. (B.) microplus*	Probable antimicrobial activity	Tanaka et al., 1999
**HEMOLYMPH CLOTTING FACTORS**					
HLS 2	44	Serpin	*H. longicornis*	Hemolymph clot formation	Imamura et al., 2005
HLSG-1	37.7	Serpin	*H. longicornis*	Hemolymph clot formation	Mulenga et al., 2001
RAS 3-4	43.2, 53.9	Serpin	*R. appendiculatus*	Hemolymph clot formation	Mulenga et al., 2003b
**BLOOD UPTAKE AND DIGESTION MODULATORS**					
HLSG-2	31.2	Serpin	*H. longicornis*	Probable blood digestion helper	Mulenga et al., 2001
HlMKI	12	Kunitz	*H. longicornis*	Probable blood digestion helper	Miyoshi et al., 2010
HLS-1	41	Serpin	*H. longicornis*	Probable blood uptake and digestion helper	Sugino et al., 2003
HlChI	6.7	Kunitz	*H. longicornis*	Probable blood digestion helper	Alim et al., 2012
RAMSP 1-3	32.3, 51.2, 49.5	_	*R. appendiculatus*	Probable blood digestion helper	Mulenga et al., 2003a
RAS-1 and -2	41.9, 42.7	Serpin	*R. appendiculatus*	Probable blood digestion helper	Mulenga et al., 2003b
AAS19	43	Serpin	*A. americanum*	Probable blood digestion helper	Kim et al., 2015
RMS-3 -6 -9 -13 -15 -16 -17 -21 -22	40-55	Serpin	*R. (B.) microplus*	Probable blood digestion helper	Tirloni et al., 2014a,b; Rodriguez-Valle et al., 2015
BmTI-A	13.5	Kunitz	*R. (B.) microplus*	Probable blood digestion helper	Sasaki et al., 2004
BmTI-D	1.6	Kunitz	*R. (B.) microplus*	Probable blood digestion helper	Sasaki et al., 2004
AamS6	42	Serpin	*A. americanum*	Probable blood digestion helper	Chalaire et al., 2011; Mulenga et al., 2013
Ixophilin	54.4	Kunitz	*I. scapularis*	Probable blood digestion helper	Narasimhan et al., 2013
**TICK DEVELOPMENT, OVIPOSITION, EGG LAYING, AND MOLTING FACTORS**					
BmTIs	6.2-18.4	Kunitz	*R. (B.) microplus*	Tick egg production and development	Tanaka et al., 1999
RMS-3	40	Serpin	*R. (B.) microplus*	Tick reproduction egg production	Rodriguez-Valle et al., 2012
RMS-6	40	Serpin	*R. (B.) microplus*	Probable role in embryogenesis	Rodriguez-Valle et al., 2012
RMS-19	40.7	Serpin	*R. (B.) microplus*	Role in tick development	Rodriguez-Valle et al., 2012
RMS-20	31.1	Serpin	*R. (B.) microplus*	Role in tick development	Rodriguez-Valle et al., 2012
RMS-21	12.5	Serpin	*R. (B.) microplus*	Probable role in embryogenesis	Rodriguez-Valle et al., 2012
RMS-22	10.7	Serpin	*R. (B.) microplus*	Probable role in embryogenesis	Rodriguez-Valle et al., 2012
RmKK	16.7	kunitz	*R. (B.) microplus*	Probable protection of undesired egg proteolysis	Abreu et al., 2014
BmTI-6	33.8	kunitz	*R. (B.) microplus*	Regulation of egg production and proteases in eggs and larvae	Andreotti et al., 2001; Sasaki et al., 2004; Sasaki and Tanaka, 2008
RsTIs	8-18	kunitz	*R. (B.) microplus*	Regulation of egg production and proteases in eggs and larvae	Sant'Anna Azzolini et al., 2003
Tick FRP	32	Kazal	*H. longicornis*	Role in tick oviposition	Zhou et al., 2006
AAS19	43	Serpin	*A. americanum*	Role in tick oviposition	Kim et al., 2016
**HOST-EXTRINSIC PATHWAY TICK INHIBITORS**					
Ixolaris	15.7	Kunitz	*I. scapularis*	Blocks FVIIa/TF complex activity	Francischetti et al., 2002
Penthalaris	35	Kunitz	*I. scapularis*	Block FVIIa/TF complex activity	Francischetti et al., 2004
BSAP1	9.3	_	*O. savignyi*	Targets tissue factor (TF)	Ehebauer et al., 2002
BSAP2	9.1	_	*O. savignyi*	Targets tissue factor (TF)	Ehebauer et al., 2002
**HOST-INTRINSIC PATHWAY TICK INHIBITORS**					
IrCPI	9.7	Kunitz	*I. ricinus*	Blocks FXII, FXI, and kallikrein activation	Decrem et al., 2009
BmTI-A	13.5	Kunitz	*R. (B.) microplus*	Blocks plasmin, elastase, and plasma kallikrein	Tanaka et al., 1999
Rhipilin-2	22	Kunitz	*R. (B.) microplus*	Affects APTT test clotting time	Cao et al., 2013
Haemaphysalin	–	Kunitz	*H. longicornis*	Blocks kallikrein-kinin system activation	Kato et al., 2005a,b
DvKPI	62	Kunitz	*D. variabilis*	Affects APTT test clotting time	Ceraul et al., 2008
**HOST-FX(a) FACTOR TICK INHIBITORS**					
TAP	7	Kunitz	*O. moubata*	Blocks FXa activity	Waxman et al., 1990
FXa inhibitor	7	Kunitz	*O.savignyi*	Blocks FXa activity	Gaspar et al., 1996
	17	_	*H. truncatum*	Blocks FXa activity	Joubert et al., 1995
	15	_	*H. dromaderii*	Blocks FXa activity	Ibrahim et al., 2001b
	65	Serpin	*R. appendiculatus*	Blocks FXa activity	Limo et al., 1991
AAS19	43	Serpin	*A. americanum*	Blocks FXa and plasmin action	Kim et al., 2015, 2016
Amblyomin-X	13.5	Kunitz	*A. cajennense*	Blocks FVIIa/TF complex activity and prothombin conversion	Batista et al., 2010; Branco et al., 2016
**HOST-THROMBIN INHIBITING TICK FACTOR**					
BmAP	60	_	*R. (B.) microplus*	Thrombin inhibitor	Horn et al., 2000
Microphilin	1.7	_	*R. (B.) microplus*	Thrombin inhibitor	Ciprandi et al., 2006
BmGTI	26	_	*R. (B.) microplus*	Thrombin inhibitor	Ricci et al., 2007
RMS 15	48		*R. (B.) microplus*	Thrombin inhibitor	Rodriguez-Valle et al., 2015; Xu et al., 2016
Boophilin	13.9	Kunitz	*R. (B.) microplus*	Thrombin inhibitor	Macedo-Ribeiro et al., 2008; Assumpção et al., 2016
Ixin	–	_	*I. ricinus*	Thrombin inhibitor	Hoffmann et al., 1991
Iris	43	Serpin	*I. ricinus*	Thrombin inhibitor	Leboulle et al., 2002b
Madanin 1	67	_	*H. longicornis*	Thrombin inhibitor	Iwanaga et al., 2003
Madanin 2	71	_	*H. longicornis*	Thrombin inhibitor	Iwanaga et al., 2003
Chimadanin	7.4	_	*H. longicornis*	Thrombin inhibitor	Nakajima et al., 2006
HLS2	44	Serpin	*H. longicornis*	Weak thrombin inhibitor	Imamura et al., 2005
Hemalin	20	Kunitz	*H. longicornis*	Thrombin inhibitor	Liao et al., 2009
IxSc-1E1	45	Serpin	*I. scapularis*	Thrombin inhibitor	Ibelli et al., 2014
Americanin	12	_	*A. americanum*	Thrombin inhibitor	Zhu et al., 1997
Amblin	17.4	Kunitz	*A. hebraeum*	Thrombin inhibitor	Lai et al., 2004
Variegin	3.7	_	*A. variegatum*	Thrombin inhibitor	Kazimírová et al., 2002
Hyalomin 1-4	8.4, 8.5, 8.2, 7.4	_	*H. marginatum rufipes*	Thrombin inhibitor	Francischetti et al., 2011; Jablonka et al., 2015
NTI 1	3.4	_	*H. dromaderii*	Thrombin inhibitor	Ibrahim et al., 2001a
NTI 2	14.9	_	*H. dromaderii*	Thrombin inhibitor	Ibrahim et al., 2001a
Rhipilin-1	18	Kunitz	*R. haemaphysaloides*	Thrombin inhibitor	Gao et al., 2011
RHS-1	41.9	Serpin	*R. haemaphysaloides*	Thrombin inhibitor	Yu et al., 2013
RHS-2	42.7	Serpin	*R. haemaphysaloides*	Thrombin inhibitor	Yu et al., 2013
Calcaratin	14.5	_	*R. B. calcaratus*	Thrombin inhibitor	Motoyashiki et al., 2003
–		_	*I. holocyclus*	Thrombin inhibitor	Anastopoulos et al., 1991
Ornithodorin	12	Kunitz	*O. moubata*	Thrombin inhibitor	van de Locht et al., 1996
Savignin	12	_	*O. savignyi*	Thrombin inhibitor	Mans et al., 2002
Monobin	15	Kunitz	*A. monolakensis*	Thrombin inhibitor	Mans et al., 2008
**HOST-IMMUNE SYSTEM MODULATION BY TICK FACTORS**					
BmSI-7	7.3	_	*R. (B.) microplus*	Elastase inhibitor	Sasaki et al., 2008
Lopsins	43-44	Serpin	*A. americanum*	Probable anti-inflammatory action	Mulenga et al., 2007
AamS6	42	Serpin	*A. americanum*	Inhibits elastase, plasmin, and chymase	Chalaire et al., 2011; Syrovets et al., 2012
AAS19	43	Serpin	*A. americanum*	Inhibits plasmin	Syrovets et al., 2012; Kim et al., 2015, 2016
Iris	43	Serpin	*I. ricinus*	Inhibits elastase-like proteases and suppresses pro-inflammatory cytokine secretion	Leboulle et al., 2002a; Prevot et al., 2009
Ipis-1	41.7	Serpin	*I. persulcatus*	Modulates CD14+ cells activation	Toyomane et al., 2016
IRS-2	41.9	Serpin	*I. ricinus*	Modulates T cell differentiation, T17 cell maturation and inhibits chymase and cathepsin G	Chmelar et al., 2011
Tryptogalinin	10.3	Kunitz	*I. scapularis*	Inhibits elastase, tryptase, plasmin, matryptase	Payne and Kam, 2004; Dai et al., 2012; Valdés et al., 2013
Tdpi	11.1	Kunitz	*R. appendiculatus*	Inhibits plasmin and tryptase	Paesen et al., 1999, 2007
RMS-3	40	Serpin	*R. (B.) microplus*	Probable interaction/modulation of B cell action	Rodriguez-Valle et al., 2012
BmTI 2 -3	17,1, 3.1	Kunitz	*R. (B.) microplus*	Inflammatory response modulation	Sasaki et al., 2004
**HOST ANGIOGENESIS MODULATION AND APOPTOSE INDUCTION BY TICK FACTORS**					
BmCI	6.5	Kunitz	*R. (B.) microplus*	Pro-apoptotic role and inhibits cell proliferation	Lima et al., 2010
Haemangin	14.1	Kunitz	*I. scapularis*	Abolishes angiogenesis and neovascularization	Islam et al., 2009
BmTI-A	13.5	Kunitz	*R. (B.) microplus*	Inhibits plasma kallikrein, plasmin, and elastase, cell proliferation and migration	Soares et al., 2012, 2016
Amblyomin-X	13.5	Kunitz	*A. cajennense*	Tumor cell cycle alteration, proteasome inhibitor, caspase cascade activation, angiogenesis repressor	Chudzinski-Tavassi et al., 2010; Morais et al., 2016
**ROLE IN TICK-BORNE PATHOGEN TRANSMISSION AND DEVELOPMENT**					
BmTI-A	13.5	Kunitz	*R. (B.) microplus*	Limits *B. bovis* proliferation in ticks	Rachinsky et al., 2007
DvKPI	62	Kunitz	*D. variabilis*	Modulates Rickettsia development	Ceraul et al., 2008
IrSPI	12	Kunitz	*I. ricinus*	Modulates *Bartonella henselae* development	Liu et al., 2014
Ixophilin	54.4	Kunitz	*I. scapularis*	Probable role in *B. burgdorferi* development	Narasimhan et al., 2013
**TICK MOLECULES TESTED IN VACCINE PROJECTS**					
RAS-1	41.9	Serpin	*R. appendiculatus*	Impacts engorgement and tick viability	Imamura et al., 2006
RAS-2	42.7	Serpin	*R. appendiculatus*	Impacts engorgement and tick viability	Imamura et al., 2006
BmTIs	6.2-18.4	Kunitz	*R. (B.) microplus*	Impacts tick viability and engorged tick weight	Andreotti et al., 2002
HLS-1	41	Serpin	*H. longicornis*	Impacts tick viability and the developmental cycle	Sugino et al., 2003
RmLTI	46	Kunitz	*R. (B.) microplus*	Impacts egg eclosion, viability, and larval hatchability	Andreotti et al., 2012

## The serine protease inhibitor family

**Figure 1:**
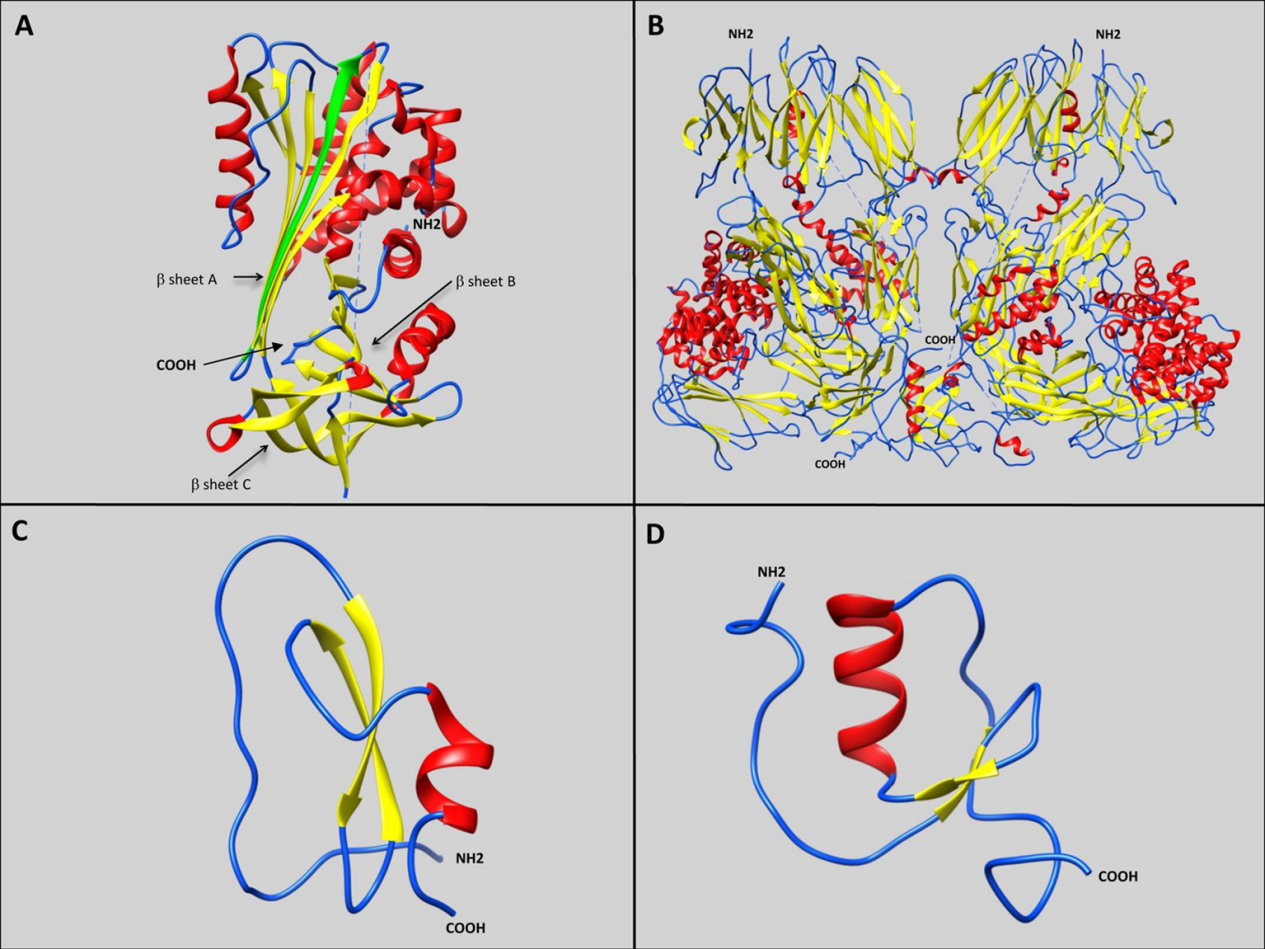
**Representative schematic diagrams of secondary structures of the four types of serine protease inhibitors: serpins, α2 macroglobulins, Kunitz-type inhibitors, and Kazal-type inhibitors**. Loops, β-sheets, and α-helices are labeled with blue, yellow, and red, respectively. **(A)** IRS 2 serpin from the *I. ricinus* tick showing the three β-sheets A, B, and C (Chmelar et al., 2011; PDB accession number: 3NDA). The green arrow represents the additional strand (s4A) formed after proteolysis, resulting in Reactive Center Loop cleavage, and insertion of the amino-terminal portion into the A β-sheet. **(B)** Human α2 macroglobulin complement component 5 in native homodimer conformation (Fredslund et al., 2008; PDB accession number: 3CU7). **(C)** Kunitz-type inhibitor Tick-Derived Protease Inhibitor (TDPI) from *R. appendiculatus* harboring one Kunitz domain composed of β-sheets and an α-helix stabilized by three highly conserved disulphide bridges (Paesen et al., 2007; PDB accession number: 2UUX). **(D)** Kazal-type inhibitor Dipetalin from blood-sucking insect *Dipetalogaster maximus*. harboring one Kazal domain composed of one α-helix with adjacent ß-sheets and loop (Schlott et al., 2002; PDB accession number: 1KMA).

Four groups of serine protease inhibitors have been identified in plants and animals, and can be classified into two main categories: trapping inhibitors including the serpins and the α2 macroglobulines (α2M); and tight-binding inhibitors including the Kunitz or Kazal domain-containing proteins (Figure 1). Trapping inhibition results in proteolytic cleavage, whereas proteases bound to tight-binding inhibitors can be released undamaged, while the inhibitors are liberated in either native or cleaved forms. Target-protease interaction occurs via the reactive center loop (RCL), which demonstrates a range of different conformations and a high degree of conformational flexibility, with each inhibitor family displaying characteristic serine protease inhibitory mechanisms. Serine protease inhibitors can be classified into two functional groups based on their ability to inhibit either trypsin or chymotrypsin: inhibitors of trypsin-like proteases such as thrombin, or inhibitors of chymotrypsin-like proteases such as elastase.

### Trapping inhibitors

#### Serpins

Serpins form a large group of homologous proteins that appear to be ubiquitous in multicellular eukaryotes. They are composed of approximately 400 amino acids and are often glycosylated. Serpins fold into an NH_2_-terminal helical domain and a COOH-terminal beta-sheet domain (Figure 1A). The serpin fold consisting of three beta-sheets (A-C) with eight or nine alpha-helical linkers and an exposed ~20 residue RCL acts as bait for the protease target (Gubb et al., 2010). An unusual aspect of serpins is their native unstable fold, where the RCL is on top and the beta-sheet A is outward. Following proteolysis however, the RCL is cleaved, and the RCL amino-terminal portion inserts into the center of beta-sheet A to form an additional (fourth) strand (s4A), which effectively stabilizes the complex structure (Law et al., 2006). The protease is thus denatured and the serpin/protease complex is targeted for degradation (Huntington et al., 2000). This amino-terminal RCL insertion can either occur upon proteolytic cleavage, or spontaneously (Huntington, 2011). Hence serpins interact with their target via a “suicide-cleavage” mechanism, resulting in the formation of an inactive covalently linked serpin/protease complex. While the majority of serpins inhibit serine proteases, they can also bind to several others such as cysteine proteases, metalloproteases, caspases (Ray et al., 1992), papain-like cysteine proteases (Irving et al., 2002), as well as some non-protease ligands, such as collagen, DNA, or protein Z. Serpins have also been ascribed several additional roles, such as heparin or heparin sulfate co-factor (Khan et al., 2011), as well as rare non-inhibitory functions; as a hormone transporter (Pemberton et al., 1988), molecular chaperone (Nagata, 1996), or tumor suppressor (Zou et al., 1994).

#### α2-macroglobulins

Members of α2-macroglobulin (α2M) group have been identified in a broad spectrum of vertebrate and invertebrate species and comprise the C3, C4, and C5 components of the vertebrate complement system (Sottrup-Jensen et al., 1985). α2Ms are considered as early-acting innate immunity components, similar to opsonin, but their role in the proteolytic attack of invading pathogens remains hypothetical. Most α2Ms are tetramers assembled from pairwise subunits with disulfide-bridges, but monomeric and dimeric forms also exist, the latter more common in invertebrates (Figure 1B) (Starkey and Barrett, 1982). Interaction with targeted proteases is initiated by proteolytic cleavage at a defined motif characterized by an exposed and highly flexible 30–40 amino acid residue region (Sottrup-Jensen, 1989). The inhibitory activity of α2Ms is directly due to their thiol-ester bond, which can be abolished by small amines such as methylamine (Larsson and Bjork, 1984). This bait region with multiple cleavage sites inhibits a broad range of proteases including serine-, cysteine-, aspartic- and metallo-proteases (Sottrup-Jensen, 1989). In addition, α2Ms could play a role as hormone transporters (Peslova et al., 2009), and can counteract inhibition from other high molecular weight inhibitors by protecting protease active sites (Armstrong et al., 1985).

### Tight-binding inhibitors

#### Kunitz/BPTI inhibitors

Initially discovered at high concentrations in beans, Kunitz proteins are typically small proteins with a molecular weight close to or less than 20 kDa (Kunitz, 1945). The most well-studied inhibitor from this family is the bovine pancreatic trypsin inhibitor (BPTI) that gives the family its name (Creighton, 1975). Generally they contain between one to twelve Kunitz domains (Laskowski and Kato, 1980), and each domain encloses disulphide-rich α helices and ß-folds stabilized by three highly-conserved disulphide bridges, leading to a compact and stable molecule (Ranasinghe and McManus, 2013) (Figure 1C). Inhibitors of this kind possess a bait region inhibiting targeted proteases that precisely matches the enzyme's catalytic site, thus generating a particularly stable substrate/inhibitor complex (Ram et al., 1954). Through the RCL region, inhibitors block the serine protease active site with a tight non-covalent interaction without any conformational changes -similarly to enzyme-substrate Michaelis complex- forming a ß-sheet between the enzyme and its inhibitor (Ascenzi et al., 2003; Krowarsch et al., 2003; Chand et al., 2004). Despite opening of the bait ring region following proteolysis, the free cut ends tend to maintain the initial fold, so the hydrolysis reaction is likely to be reversible with an equilibrium between cleaved and uncleaved [inhibitor/protease] complexes (Laskowski and Qasim, 2000). The tight-binding exposed RCL loop of Kunitz/BPTI inhibitors is suited to a wide variety of protein folds suggesting a large range of possible protease targets. However, some Kunitz domain-containing proteins with RCL region substitutions have other functions such as ion channel blockers or snake toxins (Grzesiak et al., 2000).

#### Kazal inhibitors

Initially identified in vertebrates, Kazal inhibitors have also been identified in several invertebrates (Rimphanitchayakit and Tassanakajon, 2010). They can carry from two to fifteen Kazal domains (Rawlings et al., 2004), which have between 40 and 60 amino acids of variable sequence, except for six well-conserved cysteine residues able to form three disulfide-linked sub-domains (Cerenius et al., 2010; Rimphanitchayakit and Tassanakajon, 2010). These domains comprise one α helix with three adjacent ß sheets and loops, that precisely fit the active sites of targeted proteases and block them stoichiometrically, resulting in a relatively stable protease/inhibitor complex (Figure 1D) (Laskowski and Kato, 1980). Although non-covalent binding occurs, Kazal inhibitor and protease association is tight, resulting in strong inhibition (Somprasong et al., 2006; Wang et al., 2009). Interactions between Kazal domains and proteases can occur via multiple different amino acids, thus influencing binding intensity and specificity. This enables the inhibition of several targets including trypsin, plasmin, porcine pancreatic elastase (PPE), human neutrophil elastase (hNE), chymotrypsin, proteinase K, or thrombin (Rimphanitchayakit and Tassanakajon, 2010).

## Role of serine protease inhibitors in tick biology

### Tick immune system

**Figure 2:**
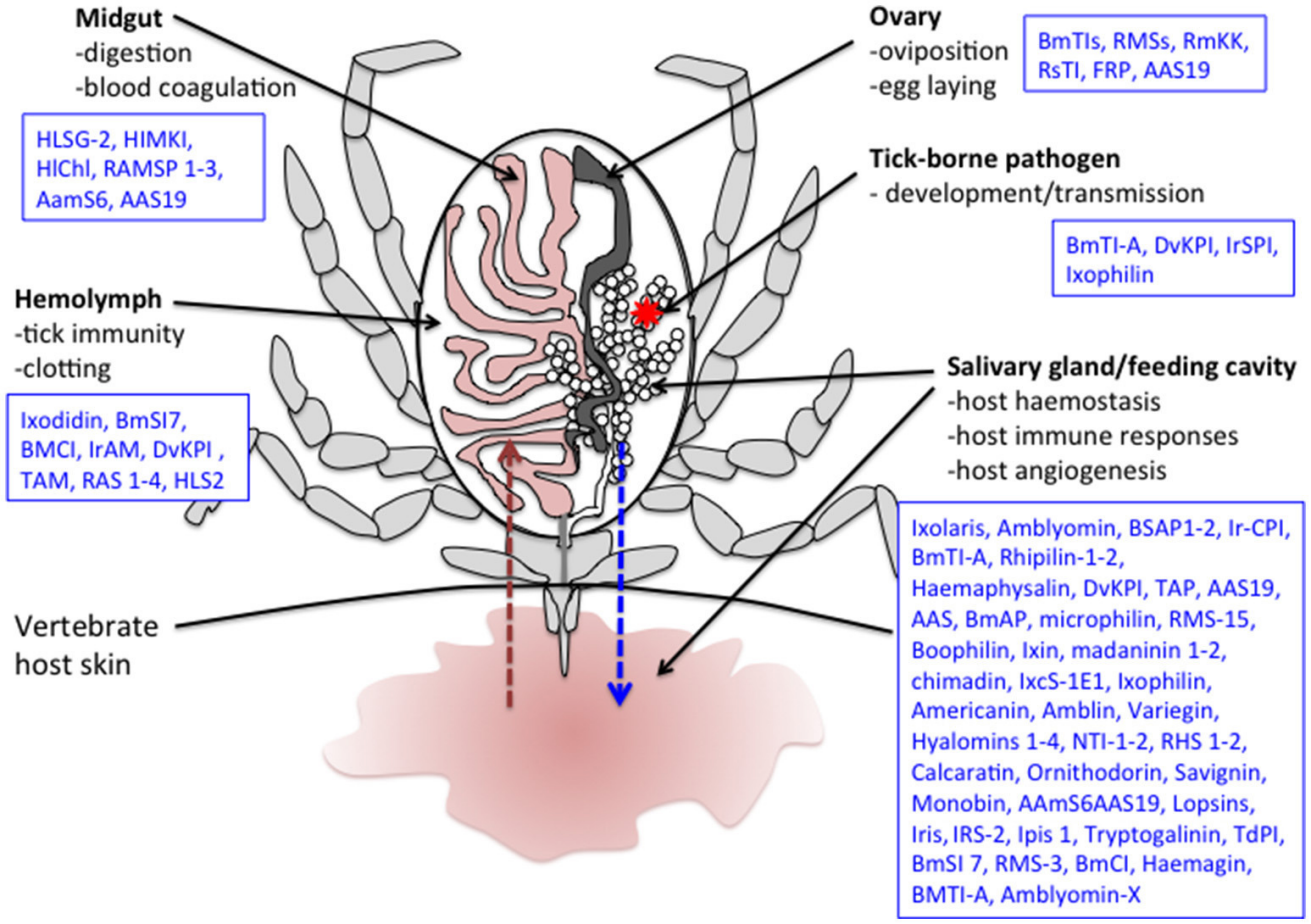
**Schematic representation of a tick during feeding**. Several tick serine protease inhibitors identified in different tick species and implicated in both tick biology/physiology and modulation of the vertebrate host responses to tick bite are also indicated. The red arrow represents blood absorption. The blue arrow represents saliva injection. The red star corresponds to a tick-borne pathogen. Note that only half of the digestive tract and a single salivary gland and ovary are represented here. *Boophilus microplus* subtilisin inhibitors (BmSI); *Boophilus microplus* Chymotrypsin inhibitor (BmCI); *Ixodes ricinus* alpha macroglobuline (IrAM); *Dermacentor variabilis* Kunitz protease inhibitor (DvKPI); Tick α-Macroglobulin (TAM); *Boophilus microplus* trypsin inhibitor (BmTIs); *R. appendiculatus* Serpins (RAS); *H. longicornis* serine proteinase genes (HLSG); *Haemaphysalis longicornis* midgut Kunitz-type inhibitor (HlMKI); *Haemaphysalis longicornis* serpin (HLS); *Haemaphysalis longicornis* chymotrypsin inhibition (HlChI); *R. appendiculatus* Midgut Serine proteinases (RAMSP); *R.B. microplus* Serpin (RMS); *Amblyomma americanum* serpin 6 (AamS6); *Amblyomma americanum* serpin 19 (AAS19); *R.B. microplus* Kunitz kallikrein inhibitor (RmKK); *I. ricinus* contact phase inhibitor (IrCPI); Tick Anticoagulant Protein (TAP); *Boophilus microplus* Anticoagulant Protein (BmAP); *B. microplus* gut thrombin inhibitor (*BmGTI*); Nymph thrombin inhibitor (NTI); *Rhipicephalus haemaphysaloides* serpin (RHS); *I. ricinus* immunosuppressive (Iris); *I. ricinus* serpin (IRS); *Ixodes persulcatus* immunosuppressive (Ipis); *I. ricinus* serine protease inhibitor (IrSPI); *R. microplus* Larval Trypsin Inhibitor (RmLTI).

It is well known that ticks possess innate immunity that also affects their vector competence (Hajdusek et al., 2013). Although all of the involved mechanisms have not yet been fully clarified, microbe phagocytosis by tick hemocytes seems to be coupled to a primitive complement-like system, a variety of antimicrobial peptides and possibly reactive oxygen species (Kopacek et al., 2010). Studies in both hard and soft ticks have implicated several tSPIs in immune responses against different microbes, mostly identified in hemolymph (Figure 2, Table 2).

**Table 2:** **Antimicrobial activities of serine protease inhibitors of ticks (tSPI)**.

**Targeted microbe**	**tSPI**	**Tick species**	**References**
*Micrococcus luteus*	Ixodidin	*R. (B.) microplus*	Fogaça et al., 2006
*Escherichia coli*	Ixodidin	*R. (B.) microplus*	Fogaça et al., 2006
*Metarhizium anisopliae*	BmSI 6, BmSI 7	*R. (B.) microplus*	Sasaki et al., 2008
	BmCI		Lima et al., 2010
*Babesia bovis*	BmTI-A	*R. (B.) microplus*	Rachinsky et al., 2007
*Chryseobacterium indologenes*	IrAM	*I. ricinus*	Buresova et al., 2009
*Rickettsia montanensis*	DvKPI	*D. variabilis*	Ceraul et al., 2007
*Bartonella henselae*	IrSPI	*I. ricinus*	Liu et al., 2014
*Borrelia burgdorferi*	Ixophilin	*I. scapularis*	Narasimhan et al., 2013

The most studied tick species in this context is *R. (B.) microplus*. Firstly, **Ixodidin**, a tSPI discovered in hemocytes, was reported to have strong inhibitory activity against *Micrococcus luteus* and, to a lesser extent against *Escherichia coli* (Fogaça et al., 2006). In addition to this bacterial clearance role, Ixodidin also possesses inhibitory activity against chymotrypsin and elastase serine proteases (Fogaça et al., 2006). However, it remains unclear whether the antimicrobial activity is due to protease inhibition or directly through peptide effects on bacterial membranes. Two additional inhibitors have been identified from *R. (B.) microplus* eggs: **BmSI 6** and **BmSI 7** (*Boophilus microplus* subtilisin inhibitors), that target Pr1 proteases from *Metarhizium anisopliae*, a fungus used as a biological insecticide (Sasaki et al., 2008). These Pr1 proteases induce host cuticle degradation, enabling hyphae penetration to obtain nutrition (Leger et al., 1987). Only *BmSI 7—*which shares several similarities with Ixodidin—has been well characterized. It harbors disulphide bonds and a trypsin inhibitor-like cysteine-rich domain (TIL), and as it is expressed in adult ovaries, midgut, SGs, hemocytes, body fat, and larvae, it could be involved in tick defense mechanisms, and/or in avoiding tick tissue degradation (Sasaki et al., 2008). Random sequencing of a tick body fat cDNA library enabled the discovery of, **BmCI** (*Boophilus microplus* chymotrypsin inhibitor), a chymotrypsin inhibitor that belongs to the Kunitz/BPTI inhibitor family (Lima et al., 2010). BmCI strongly and specifically inhibits chymotrypsin, and also hNE with reduced specificity. *BmCI* gene expression analysis demonstrated higher expression in hemocytes and ovaries than in SGs and body fat. Following infection with *M. anisopliae*, increased BmCI expression was only observed in tick hemocytes clearly suggesting a role in the tick defense system (Lima et al., 2010). Finally, among BmTIs (*Boophilus microplus* trypsin inhibitors) identified in both larvae and eggs (Tanaka et al., 1999), a double Kunitz-containing inhibitor, **BmTI-A**, had increased transcript levels in ovaries following *Babesia bovis* infection (normally transmitted transovarially in ticks), also suggesting a probable role in the tick immune system (Rachinsky et al., 2007).

**IrAM** (*I. ricinus* alpha macroglobuline), was identified in the hemolymph of *I. ricinus* as a α2M composed of two non-covalently linked subunits (Buresova et al., 2009). Alternative splicing occurring during IrAM synthesis generates seven bait variants, increasing the spectrum of targeted proteases. IrAM transcripts are detected throughout all tick developmental stages. Gene expression was higher in SGs from partially engorged females than in hemocytes or ovaries, even though IrAM protein is only detected in tick hemocytes. RNAi experiments revealed that IrAM is not involved in tick fitness, mortality, or fecundity (Buresova et al., 2009). However, IrAM enhanced phagocytosis and elimination of the bacteria *Chryseobacterium indologenes* due to its active thioester bonds, which was not observed with other bacteria such as *B. burgdorferi* (Buresová et al., 2006; Buresova et al., 2009). IrAM likely interacts with the major *C. indologenes* virulence factor, a metalloprotease, suggesting a role in the tick immune system during phagocytosis of metalloprotease-producing bacteria (Buresova et al., 2009).

Following the discovery of over-expressed genes in *Dermacentor variabilis* tick body fat and midgut in response to *Rickettsia montanensis* infection (Ceraul et al., 2007), several Kunitz/BPTI inhibitors were identified including **DvKPI** (*Dermacentor variabilis* Kunitz protease inhibitor with five Kunitz domains) (Ceraul et al., 2008). DvKPI was up-regulated six-fold in infected ticks that had fed for three days. These results suggested that DvKPI is involved in tick responses to Rickettsia, avoiding massive colonization which would be detrimental to ticks.

Several tSPIs have also been identified in *Ornithodoros moubata* soft tick hemolymph, including **TAM**, (tick α-macroglobulin), the second most abundant protein after vitellogenin (Kopacek et al., 2000). TAM is a tetrameric glycosylated protein that displays similar structural features present on IrAM, and exerts inhibitory activity against both trypsin and thermolysin (Kopacek et al., 2000). Comparing the conserved cysteine motifs between human and limulus α2Ms enables the prediction of disulfide bridge patterns which explain the atypical molecular arrangement of the four TAM bait region variants, likely arising from alternate splicing (Saravanan et al., 2003). While TAM was initially detected in tick hemocytes, significant up-regulation has also been reported in SGs and gut after 1 day of feeding (Saravanan et al., 2003). TAM is believed to be involved in tick defense systems, but an additional role as an anti-coagulant has also been postulated (Keller et al., 1993; van de Locht et al., 1996).

### Hemolymph clotting formation

As for vertebrates, effective clotting is critical in ticks to limit hemolymph loss and to inhibit pathogens from entering into the tick through the wound. Little is known about the proteins involved in tick hemolymph clotting but several tSPIs have been implicated in this defense system due to homology with known proteins (Figure 2).

Four *Rhipicephalus appendiculatus* tick serpins have been identified as potential clotting enzymes involved in the hemolymph coagulation cascade: *R. appendiculatus* serpin-1, -2, -3, and -4 (**RAS-1, -2, -3, and -4**) (Mulenga et al., 2003b). All exhibit similarities ranging from 25 to 30% with limulus intracellular coagulation inhibitor type 1 (LICI-1) from the Japanese horseshoe crab *Tachypleus tridendatus*. RAS-3 harbors also amino acids similarities with LICI-2 and is comparably expressed in all tick organs, while RAS-1, -2 and -4 expression was stronger in SGs than in other tick organs (Mulenga et al., 2003b).

**HLS2**, a serpin from *Haemaphysalis longicornis*, contains an RCL with high sequence similarities to both vertebrate and invertebrate serpins, and may interact with both chymotrypsin and thrombin (Imamura et al., 2005). HLS2 also demonstrates similar molecular features to RAS-3 and LICI, and its mRNA has only been detected in the hemolymph of partially or fully engorged nymphs and females, suggesting a possible role in endogenous hemolymph circulation. In the same tick species, the serpin **HLSG-1** harbors high similarity (32–44%) to Japanese and mangrove horseshoe crab clotting factor C precursors, mouse manan-binding lectin serine protease 1, and rat/mouse hepsin proteins (Mulenga et al., 2001). In partially fed ticks HLSG-1 transcripts were weakly expressed in the midgut and strongly detected in the SGs.

### Blood uptake and digestion modulation

As strict haematophagous acari, ticks require blood meals to complete their development and reproduction. These arthropods are pool-feeders and create haemorrhagic pools from which they collect nutritive fluids while biting, and interestingly, some female hard ticks can imbibe enough blood to increase in size by as much as 100 times (Sonenshine and Anderson, 2014). This unique feeding method implies the existence of very effective blood uptake and digestion mechanisms, in which several tSPIs have now been implicated (Figure 2).

In 2001, Mulenga et al. identified **HLSG-2** in *H. longicornis* ticks, a serpin with chymotrypsin-like protease selectivity (Mulenga et al., 2001). HLSG-2 transcripts were only detected in partially fed ticks and expression increased in parallel with feeding duration. HLSG-2 expression was strong in midgut and weak in salivary glands, suggesting probable links with blood uptake and digestion processes (Mulenga et al., 2001). In the same tick species, another serpin-1 (**HLS-1**) harboring similarities to the *I. ricinus* SG serpins RAS-2 and RAS-1 (Sugino et al., 2003) was identified. Taking into account additional sequence homologies with other anticoagulation factors, its specific expression in midgut of partially fed ticks, as well as the fact that clotting time can be delayed by HLS-1 in a dose-dependent manner, all indicate that HLS-1 has a probable role in tick blood meal uptake as well as maintaining blood fluidity in the midgut (Sugino et al., 2003). Then in 2010, Miyoshi et al. identified a Kunitz-type tSPI exclusively expressed in the midgut of adult ticks named **HlMKI** (*Haemaphysalis longicornis* midgut Kunitz-type inhibitor) (Miyoshi et al., 2010). Immunofluorescent analysis demonstrated that HlMKI likely interacts with HlSP, a hemolytic serine protease expressed in the tick midgut, and that both proteins harbored similar expression patterns with a 72 h peak during feeding (Miyoshi et al., 2007). HlMKI displayed inhibitory activity against the HlSP protein, and against chymotrypsin and elastase to a lesser extent. Altogether these results suggested that HlMKI can regulate blood digestion in tick midgut via HlSP modulation (Miyoshi et al., 2010). Lastly, another Kunitz-type tSPI was identified in *H. longicornis* hemocytes, and was named **HlChI** because of its chymotrypsin inhibitory profile (Alim et al., 2012). HlChI has strong chymotrypsin inhibitory activity but low trypsin inhibitory activity. The *HlChI* gene is expressed in larvae, nymphs, and adults during all feeding phases, and transient up-regulation has been clearly detected from feeding initiation to repletion. During feeding, HlChI is mainly localized in hemocytes, though low expression levels were also detected in midgut, salivary glands, ovaries, and the epidermis (Alim et al., 2012). Several HlChI-RNAi-injected ticks died 48 h after feeding, while others ingested significantly smaller and slower blood meals, laid fewer eggs, and demonstrated lower larvae conversion (Alim et al., 2012). During feeding, HlChI expression in hemocytes peaks at 96 h, coinciding with low proteolytic activity and a low homeostasis level maintained by a few principal inhibitors (Franta et al., 2010; Alim et al., 2012). Thus HlChI is likely an indirect but essential actor in both vital blood feeding and tick reproduction processes.

In *R. appendiculatus* ticks, three serine protease inhibitors of chymotrypsin or trypsin and named **RAMSP 1-3** (*R. appendiculatus* midgut serine proteinases 1-3) were identified, and are likely involved in feeding processes or blood digestion (Mulenga et al., 2003a). RAMSP-1 and -2 transcripts have only been detected in partially fed ticks, while RAMSP-3 mRNA was detected in both unfed and partially fed ticks, with stronger signals in the latter. RAMSPs expression is not restricted to the midgut because RAMSP-1 is equivalently expressed in all tick organs, while RAMSP-2 is weakly expressed in both SGs and midgut, and RAMSP-3 is more weakly expressed in SGs than midgut (Mulenga et al., 2003a). Additionally, all three RAMSPs are expressed in tick carcasses (whole tick without SGs and midgut), suggesting widespread distribution of these inhibitors in other tick tissues (Mulenga et al., 2003a). In terms of **RAS** serpins, RAS-1 and RAS-2 mRNAs can be detected at all life stages, as well as in both sexes, with positive detection in 4-day partially fed and fully engorged ticks (Mulenga et al., 2003b). This suggests gene expression both during and after feeding, although they were not expressed in saliva, likely because they do not contain signal peptide sequences (Mulenga et al., 2003b). Indeed, RAS-1 and RAS-2 might be associated with feeding in both SGs and the midgut by modulating blood uptake and digestion. Confirmation occurred when significantly fewer fully engorged nymphs were counted when ticks were fed on vaccinated compared to non-vaccinated cattle (Imamura et al., 2006).

Then in 2014, NGS technologies enabled the identification of 22 **RMS** serpins (RMS-1 to RMS-22) from *R. (B.) microplus* (Tirloni et al., 2014a,b), among which 18 full-length coding sequences were identified (Rodriguez-Valle et al., 2015). While serpin consensus patterns were conserved, these 18 members of the RMS family showed high amino acid sequence variability suggesting a broad spectrum of targeted serine proteases. Transcription levels of RMS-13, -15, -16 in SGs, or RMS-6, -7, -9, -17 in both SGs and the midgut, suggested that they play a role in the blood meal process, either in uptake or during digestion (Rodriguez-Valle et al., 2015). Functionally, RMS-3 strongly inhibits chymotrypsin and elastase, but only weakly trypsin and thrombin. RMS-15 is a strong thrombin inhibitor and RMS-6 a chymotrypsin inhibitor. Finally while RMS-21 and -22 are not secreted, they were detected in the midgut and SGs suggesting a probable role in proteolysis activity during blood digestion (Rodriguez-Valle et al., 2015). Among the previously mentioned *R. (B.) microplus* BMTIs, the Kunitz inhibitors **BmTi A** and **BmTI D** were also believed to play an important role in feeding by inhibiting human prekallikrein (HuPK) implicated in the coagulation cascade (Sasaki et al., 2004), thus facilitating blood fluid uptake.

**Figure 3:**
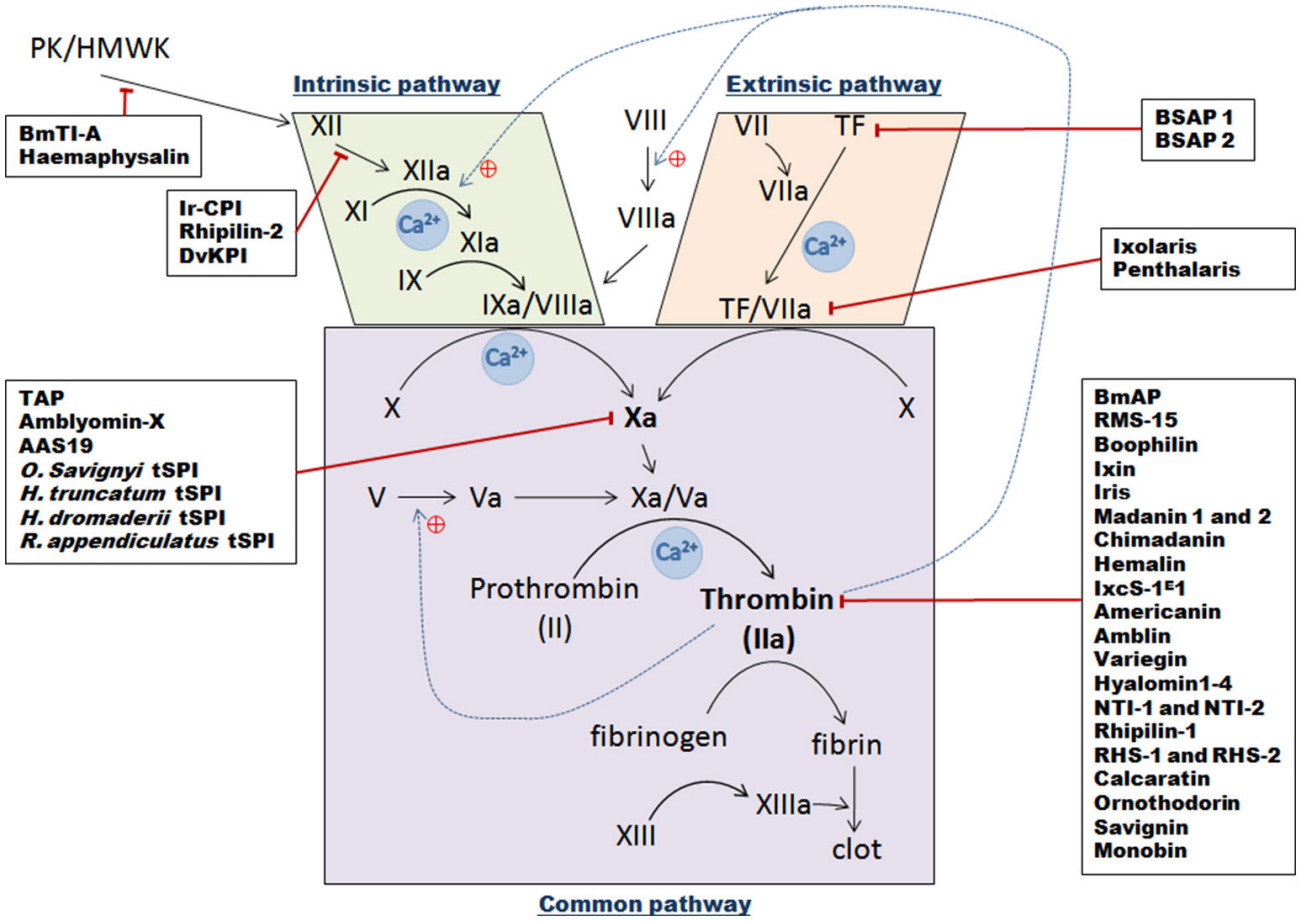
**Schematic overview of the three vertebrate blood coagulation cascade pathways, with indicated tick serine protease inhibitors and their targets**. The intrinsic pathway is activated following a trauma and when blood comes into contact with subepithelial cells [contact phase with XII, Prekallikrein (PK), and High Molecular Weight Kininogen molecules (HMWK)], which then results in successive activation of factors XII, XI, and IX. The IXa/VIIIa complex then activates transformation of factor X into factor Xa. The extrinsic pathway is initiated following contact between Tissue Factor (TF) from vessels and circulating factor VII. After injury, they form a complex and catalyze the activation of factor X into factor Xa. Once activated by either pathway, factor Xa complexes with its cofactor Va to form the pro-thrombinase complex, which can then convert prothrombin into thrombin. Thrombin then transforms fibrinogen into fibrin to create a plug. Black arrows represent direct activation, dotted blue lines show positive thrombin feedback, red lines correspond to tSPI inhibition targets.

The serpin **AamS6** was identified in the *Amblyomma americanum* tick (Mulenga et al., 2013). Both AamS6 mRNA and protein are strongly expressed in the SGs and midgut in unfed ticks, as well as during the first 72 h of feeding, before fading at 96 h, suggesting injection into the bite site and a role in tick anchorage to the host (Chalaire et al., 2011). As expected according to its serpin-like sequences, AamS6 interacted clearly and selectively with trypsin, chymotrypsin, elastase, and chymase, but also surprisingly with papain-like cysteine proteases, indicating that it is a cross-class protease inhibitor (Mulenga et al., 2013). In addition, AamS6 seems to transiently interact with fibrin-lysing plasmin. Despite only *in vitro* data providing the proof for plasmin-AamS6 interaction, it was hypothesized that this inhibitor could sustain blood flow to the tick feeding site and prevent clot formation (Mulenga et al., 2013). In this series of experiments, AamS6 only delayed recalcification time (RCT, the time to plasma clotting once calcium ions and blood-clotting co-factor(s) are reintroduced to citrate plasma), and did not inhibit any of the three coagulation pathways (extrinsic, intrinsic, and common, Figure 3). Platelet aggregation inhibition was also reported, that, when combined with previous results, bears out AamS6's involvement in inhibiting blood coagulation both in the midgut and via saliva secretion (Mulenga et al., 2013). Later, an *A. americanum* transcriptomic study identified a novel serpin, **AAS19**, which has the most fully conserved RCL among the ixodid ticks (Kim et al., 2015; Porter et al., 2015). The presence of the tripeptide Arg-Gly-Asp which constitutes a “RGD” motif, in the AAS19 sequence suggests a potential relationship between integrin GPIIb-IIIa and AAS19 during platelet aggregation (Nurden, 2014). Additionally, as AAS19 is abundantly and mostly expressed in the midgut at 96 h during feeding, it likely ensures that host blood doesn't clot during feeding (Porter et al., 2015). AAS19 demonstrates inhibitory activity against plasmin, FXa, and FXIa, and at a lower efficacy rate against FXIIa, FIIa (activated thrombin), FIXa, and tryptase, demonstrating its broad activity spectrum. In addition, the three coagulation pathways have delayed clotting in the presence of AAS19, likely due to weak thrombin inhibition. Altogether these results support AAS19 involvement in blood digestion (Kim et al., 2015).

Finally, **Ixophilin** was identified as a Kunitz thrombin inhibitor secreted in the midgut of *I. scapularis* and which shares homology with Hemalin and Boophilin (see below) (Narasimhan et al., 2013). Ixophilin was preferentially expressed in adult and nymphal midgut and was induced upon feeding, consistent with a potential role in preventing blood clotting in the midgut. In addition, Ixophilin mice immunization experiments demonstrated that ixophilin was necessary for efficient engorgement (Narasimhan et al., 2013).

### Tick development, oviposition, egg laying

Tick oviposition and egg laying are essential aspects of the tick life cycle determining tick population expansion. These processes are regulated by many proteins, including tSPIs (Figure 2). Inhibitors may be endogenous to certain organs and, for ovaries, it appears that some proteins—including tSPIs—can be captured from the midgut by receptor-mediated endocytosis followed by incorporation into the eggs (Tufail and Takeda, 2009).

Several tSPIs named **BmTIs**, with a similar target spectrum (trypsin, plasmin, and HuPK), have been discovered in the eggs and larvae of *R. (B.) microplus* (Tanaka et al., 1999). However, differing concentrations and inhibitor specificity changes have been reported between egg and larval stages (Andreotti et al., 2001; Sasaki et al., 2004). Andreotti et al. showed a 69.7 and 71.3% reduction in both engorged tick number and egg weight respectively when female ticks were fed on BmTIs-immunized cattle, confirming a crucial role for BmTIs in egg production and development (Andreotti et al., 2002). **BmTI-6**, a Kunitz tSPI identified in ovaries, was also expressed in tick body fat and demonstrated specific inhibitory activity against trypsin and trypsin-like proteases, such as plasmin (Sasaki and Tanaka, 2008). As for other BmTIs, BmTI-6 is suspected to play a role in controlling endogenous proteases in the egg and larval stages (Andreotti et al., 2001; Sasaki et al., 2004).

The **RMS-3** serpin is expressed in the SGs of semi-engorged females, but lower expression levels were also observed in the midgut and ovaries of *R. (B.) microplus* (Rodriguez-Valle et al., 2012). *In vitro* feeding assays showed that both egg weight and larval transformation rates were reduced in female ticks pre-fed on anti-RMS-3 sheep serum, thus implicating RMS-3 in reproduction and egg development (Rodriguez-Valle et al., 2012). An additional Kunitz serine protease inhibitor with trypsin and kallikrein inhibitory activities named **RmKK** was recently discovered in *R. (B.) microplus* eggs (Abreu et al., 2014). Although the native protein was obtained from eggs, the RmKK transcript was only detected in the midgut, suggesting possible midgut expression and subsequent transport to the ovaries and egg incorporation. Interestingly, kallikrein inhibitors were thought to protect against undesired egg proteolysis (Willadsen and Riding, 1980). Lastly, four other serpins were also recently implicated in tick embryogenesis regulation or vitellogenesis: **RMS-19** and **RMS-20**, which are expressed in all tissues and at all stages, **RMS-6** which is only detected in ovaries, and **RMS-21 and -22** which are only detected in eggs (Rodriguez-Valle et al., 2015).

Studies on the dog tick *Rhipicephalus sanguineus* identified a group of proteins belonging to the Kunitz/BPTI tSPI family, called **RsTIs** in larval stages (Sant'Anna Azzolini et al., 2003). Three of which—**RsTIQ2, RsTIQ7** and **RsTIS5—**inhibit trypsin, neutrophil elastase, and human plasmin. RsTIQ2, and to a lesser extent RsTIQ7, also inhibit HuPK (Sant'Anna Azzolini et al., 2003). Because RsTIs are similar in structure and inhibitory activity to previously described tSPIs from *R. (B.) microplus*, a similar role in egg production has been hypothesized.

A human follistatin-related-protein (**FRP**) homolog was also identified in *H. longicornis* ticks, and is implicated in tick oviposition (Zhou et al., 2006). This protein harbors three distinct domains, a follistatin-like domain, a Kazal domain and two EFh calcium–binding motifs. Polyclonal antibodies revealed FRP presence in tick salivary glands, midgut, body fat, hemocytes, and a strong expression in ovaries (Zhou et al., 2006). RNAi experiments silencing FRP in adult rabbit-fed ticks as well as the use of anti-FRP antibodies showed significant negative effects on tick oviposition while no differences were observed in feeding duration, engorgement weight, and survival (Zhou et al., 2006).

Finally, when the aforementioned *A. americanum* **AAS19** gene was RNAi silenced, ticks imbibed much less blood and presented curious body deformities compared to controls, likely due to deficiencies in hemostasis regulation (Kim et al., 2016). In addition, ticks that fed on rAAS19-immunized rabbits took smaller blood meals and detached prematurely. Following a second round of infestation on these rabbits, ticks also failed to lay eggs, suggesting an important role for AAS19 in both tick homeostasis and reproduction (Kim et al., 2016).

## Roles of serine protease inhibitors in modulating vertebrate host responses

The long feeding period of ticks necessitates extended control over the vertebrate host's haemostasis and immunity. During this feeding process, ticks alternatively inject saliva into and then absorb fluids from the bite wound. To enable the feeding process and avoid tick rejection, several salivary components are thought to control host responses, including several tSPIs (Figure 2).

### Anti-hemostatic effects of tick serine protease inhibitors

Hemostasis in vertebrates is a tightly regulated process to avoid blood leakage following injury (Aird, 2003). Many biochemical mechanisms are involved in blood clot formation with a central enzymatic cascade that can be activated by three different pathways (Figure 3). When the vessel epithelium is damaged, tissue factors (TF) expressed by epithelium cells initiate clot cascades, leading to activation of extrinsic blood coagulation pathways. Displaying TF triggers factor VII activation thus forming TF/VIIa complexes which then activate central factor X. The second intrinsic pathway is activated by contact factors—XII, prekallikrein (PK) and high molecular weight kininogen (HMWK)—that cause successive activation of intermediate factors (factors XI, IX), and IXa/VIIIa complex formation, which also activates factor X. Thus intrinsic and extrinsic coagulation pathways merge in order to activate Factor X to Xa. Then in the “common pathway,” activated factor Xa interacts with its co-factor Va and forms the pro-thrombinase complex. This complex then processes pro-thrombin (factor II) into thrombin (factor IIa) that converts fibrinogen into fibrin which then polymerizes to constitute a clot. Ticks prevent clot formation in the host, in the micro hematoma at the bite site, but also, in tick mouthparts and midgut. They inject numerous proteins via their saliva into the blood bowl, mostly tSPIs that affect serine proteases or their zymogens (Factors II, VII, IX, X, XI, XII, and prekallikrein) implicated in clot formation (Tatchell, 1969; Ribeiro et al., 1985). As anticoagulant factors, these inhibitors are essential in allowing ticks to take their blood meal, and concomitantly ingest and transmit pathogens. In addition, during the long feeding period, the blood in the tick midgut is maintained in a fluid state until repletion. To retain blood fluidity, anticoagulants are secreted from the tick midgut epithelium to the midgut lumen. Coagulation inhibitors secreted into the midgut mostly inhibit thrombin whereas anticoagulants secreted from SGs mainly inhibit FXa.

#### Extrinsic pathway tick inhibitors

*I. scapularis* was intensively studied in order to understand tick saliva anticoagulant properties, leading to the discovery of multiple anticoagulant molecules, the first being **Ixolaris** from the SGs (Francischetti et al., 2002). This inhibitor, similar to human hTFPI (tissue factor pathway inhibitor), contains two Kunitz domains. Ixolaris, like hTFPI, either blocks FVIIa/TF complex activity through direct interaction with the active site, or sterically via the formation of a tight complex [FVIIa/TF/Ixolaris/FX(a)], resulting in effective extrinsic pathway inhibition (Francischetti et al., 2002). Ixolaris, reaching high concentrations only in the feeding cavity *in vivo*, behaves as a fast ligand of the FX and FXa heparin binding exosite (HBE)—a site distinct from the FXa reactive site—presumably via its second atypical Kunitz domain (Monteiro et al., 2005). The prothrombinase complex could be resistant to physiological concentrations of Ixolaris due to competition with its prothrombin substrate, as reported for hTFPI (Mast and Broze, 1996). Ixolaris' mechanism of action was then proposed to be competitive and concentration dependent (Monteiro et al., 2005). Further analyses of an SG cDNA library enabled the discovery of another Kunitz domain protein implicated in coagulation inhibition, **Penthalaris** (Francischetti et al., 2004). This inhibitor harbors five tandem Kunitz domains and like Ixolaris, inhibits FVIIa/TF-induced FX activation at high concentrations by binding to the HBE of both FX and FXa.

Finally, two *Ornithodoros savignyi* saliva anti-coagulants have been identified: **BSAP1** and **BSAP2** (Ehebauer et al., 2002). These proteins do not inhibit the intrinsic pathway, whereas the extrinsic pathway appeared to have delayed clot formation. Therefore, these proteins may only target TF, as FVII does not seem to be inhibited. It may be the first time a direct TF-interacting protein has been reported, because until now the majority of known extrinsic pathway inhibitors target other factors forming complexes with TF.

#### Intrinsic pathway inhibitors

The Kunitz protein **Ir-CPI** was identified in *I. ricinus* SGs and binds to contact phase factors, FXII, FXI, and kallikrein (Decrem et al., 2009). *In vitro* experiments showed that Ir-CPI considerably prolonged aPTT, without modifying the extrinsic pathway, whereas experiments on both venous and arterial thrombus formation in animal models showed that Ir-CPI mainly inhibits clot propagation and thrombin generation (Decrem et al., 2009). Like Ixolaris, Ir-CPI does not block amidolytic activity of targeted proteases by binding to the catalytic site as is usual for Kunitz-type inhibitors, but likely acts by binding to an exosite, thereby preventing enzyme activity with high affinity steric hindrance (Laskowski and Kato, 1980).

Among the BmTIs from *R. (B.) microplus* tick eggs and larvae, **BmTI-A** strongly inhibits trypsin, hNE, plasmin and HuPK (Tanaka et al., 1999). It was initially described with two Kunitz domains, the first implicated in trypsin and HuPK inhibition and the second inhibiting hNE (Tanaka et al., 1999; Guerrero et al., 2005). Subsequently, a further five Kunitz-BPTI domains were identified (Soares et al., 2016). BmTI-A transcripts are mainly expressed in the tick midgut, and are weakly expressed in SGs and ovaries (Tanaka et al., 1999). It is possible that the inhibitor is transferred from ovaries to the larval stage where it could be important for controlling blood coagulation, inflammation, and angiogenesis during the larval feeding process, by inhibiting plasma kallikrein, neutrophil elastase, and plasmin (Tanaka et al., 1999; Soares et al., 2016).

Another Kunitz-type tSPI with a unique Kunitz domain named **Rhipilin-2** was identified in *Rhipicephalus hemaphysaloides*, and is highly similar to members of the TFPI mammalian protein family (Cao et al., 2013). Rhipilin-2 does not prolong recalcification time in PT assays, but increases coagulation time of citrated rabbit plasma in aPTT assays. It inhibits approximately 60% of trypsin activity but does not inhibit thrombin. Rhipilin-2 gene expression was only detected in SGs and the midgut of fed ticks (Cao et al., 2013). One-day-fed ticks presented the highest expression that gradually faded until total engorgement, suggesting probable injection via saliva into the host during feeding to restrict blood clotting at the wound site (Cao et al., 2013).

**Haemaphysalin**, a Kunitz-type inhibitor from the hard tick *H. longicornis* also inhibits intrinsic coagulation pathways by blocking kallikrein-kinin system activation (Kato et al., 2005a,b). Its acts via its two Kunitz domains, and does not affect the amidolytic activities of intrinsic coagulation factors. Direct binding assays demonstrated binding of the COOH-terminal domain to both high molecular weight kininogen (HMWK) and factor XII (Kato et al., 2005a,b). The COOH-terminal domain may then inhibit factor XII and HMWK association on the cell surface, and hence inhibits kallikrein-kinin system activation by interfering with prekallikrein and factor XII reciprocal activation. Zn^2+^ ions appear to be involved in interactions between haemaphysalin and its targets, suggesting that these cations induce conformational changes which enable haemaphysalin's inhibitory effect (Kato et al., 2005a,b).

Previously mentioned studies of **DvKPI** highlighted its ability to inhibit the coagulation cascade as revealed by both delayed aPTT assays and robust antitrypsin activity. Although DvKPI expression was detected in both body fat and SGs, the highest expression was in the midgut and which increased upon feeding, demonstrating that its anticoagulant activity in the midgut is essential (Ceraul et al., 2008).

#### FX(a) factor inhibitors

Tick anticoagulant protein **TAP**, the first tSPI to specifically inhibit FX(a) factor was identified in the soft tick *Ornithodoros moubata* as a Kunitz inhibitor (Waxman et al., 1990). Whereas Kunitz inhibitors are generally highly basic, TAP is acidic, and was classified as a slow, tight-binding inhibitor, because it requires at least 15-min pre-incubation for maximal FX(a) inhibition.

**Amblyomin-X** was initially discovered following SG transcriptome sequencing of the *Amblyomma cajennense* Cayenne tick (Batista et al., 2010). This protein harbors one Kunitz-type domain, and is observed in monomeric, dimeric, trimeric, and tetrameric conformations. Amblyomin-X inhibits FXa in a non-competitive manner but also increases PT and aPTT, suggesting an effect on prothrombin conversion (Batista et al., 2010; Branco et al., 2016). Four hypotheses have been proposed to explain the FXa inhibitory mechanism of Amblyomin-X. Firstly, as Amblyomin-X shares amino acid sequence similarities with TAP, it could bind FXA as a slow, tight-binding inhibitor. The second is that the strongly negatively charged COOH-terminal domain of Amblyomin-X could bind to positively charged FXa exosites. Thirdly, an additional binding of the Amblyomin-X NH_2_-terminus to the FXa active site could exist. Lastly, Lys30 from the characteristic Kunitz domain loop could bind to the FXa active site (Batista et al., 2010).

The aforementioned **AAS19** serpin from *A. americanum* also appears to be injected into the host during feeding, and enhances feeding success by inhibiting trypsin-like proteases including Fxa, hemostasis, and host immune-defenses (Kim et al., 2015, 2016).

Lastly, and despite not being fully characterized, several other FX(a) inhibitors from different tick species have been reported. Amongst these is an SG protein from the soft tick *O. savignyi*, with an approximate 7 kDa molecular weight and six cysteine residues suggesting a single Kunitz domain. FXa inhibition appears specific, although thrombin was also very weakly inhibited (Gaspar et al., 1996). In *Hyalomma truncatum*, the bont-legged tick, several SG proteins inhibiting both extrinsic and intrinsic coagulation pathways were detected (Joubert et al., 1995). Of these, one 17 kDa nameless protein possesses Factor Xa inhibitory activity and was only identified in females pre-fed for 5–7 days, suggesting involvement in tick feeding process. This FXa inhibition appeared to be non-competitive, in contrast to TAP from *O. moubata*, but similar to another 15 kDa tSPI identified in nymphs of the camel tick, *Hyalomma dromedarii*. This last tSPI totally inhibits FXa but only partially inhibits thrombin activity (30% inhibition). Extremely efficient FXa inhibition could be explained by the presence of two binding sites on the inhibitor (Ibrahim et al., 2001b). Finally, a 65 kDa FXa inhibitor from *R. appendiculatus* was also isolated from SG extracts (Limo et al., 1991). No complexes between FXa and this inhibitor were identified, and it was established that inhibition may occur via exosite binding.

#### Thrombin inhibitors

Crude saliva of *R*. (*B.) microplus* was initially investigated because of its effective bovine plasma coagulation inhibiting properties, and subsequently several thrombin inhibitors were identified. **BmAP** was described as a non-tight binding thrombin inhibitor, possibly dimerized, and which interacts with both thrombin active sites and subsites (Horn et al., 2000). Two **microphilin** isoforms were then discovered, and which are the smallest non-tight binding thrombin inhibitors identified thus far (Ciprandi et al., 2006). Microphilin only interacts with thrombin at exosite I, the crucial site for both fibrinogen and platelet thrombin receptor interactions. **BmGTI**, from the *R. (B.) microplus* midgut, was reported to inhibit fibrinogen cleavage by thrombin (Ricci et al., 2007), via interaction with thrombin's positively charged exosite I (Monteiro, 2005). The serpin **RMS-15** was identified as the highest affinity thrombin inhibitor (Rodriguez-Valle et al., 2015; Xu et al., 2016). RCT assays showed that plasma clotting time is delayed in the presence of RMS-15, in a dose-dependent manner. The elevated RMS-15 IgG titres found in bovine sera after prolonged exposure to tick infestation, and the presence of RMS-15 transcript in SGs and midgut (Rodriguez-Valle et al., 2015), suggest a likely secretion of this serpin into the tick-feeding site, which then acts on host coagulation (Xu et al., 2016). Lastly, a Kunitz-type serine-protease inhibitor harboring two Kunitz domains was isolated from the tick midgut and named **Boophilin** (Macedo-Ribeiro et al., 2008). Boophilin greatly increases TT (Assumpção et al., 2016), significantly affects PT, and increases aPTT, albeit weakly (Macedo-Ribeiro et al., 2008). Boophilin displayed partial effects on plasma kallikrein and weak inhibitory effects on FVIIa, showing probable, but limited, implication in both extrinsic and intrinsic pathways. In addition, boophilin can block amidolytic activity of other trypsin-like serine proteases, most notably trypsin and plasmin (Macedo-Ribeiro et al., 2008). Boophilin is a strong thrombin inhibitor forming a stable thrombin/boophilin complex in a non-canonical manner (Macedo-Ribeiro et al., 2008). The proximal interaction between boophilin and meizothrombin (MzT) suggests that boophilin may not only target circulating thrombin but also the MzT intermediate. When boophilin and MzT are complexed, the boophilin NH_2_ terminus remains effectively available and able to bind proximal coagulation factors including FXa.

At the end of the nineteenth century it was demonstrated that *I. ricinus* saliva also contains anticoagulant molecules (Sabbatini, 1899). **Ixin**, a specific thrombin inhibitor, was isolated from adult saliva in 1991 (Hoffmann et al., 1991), and the serpin **Iris—**for “*I. ricinus* immunosuppressor”—was identified in 2002 (Leboulle et al., 2002b). Iris expression is induced in SGs while ticks feed, peaking at day four, coinciding with the period when the *I. ricinus* female ingests the most amount of blood. Iris demonstrates dose-dependent FXa inhibition and inhibits close to 30% of thrombin. Its inhibitory activity arises from the RCL domain where the P1 residue plays a key role, which was confirmed with Iris structural model interactions (Prevot, 2006). Iris hampers fibrinolysis by inhibiting both tissue plasminogen activator (t-PA) and elastase released by leukocytes. It also acts as a hypo-fibrinolytic factor by targeting serine proteases, especially elastase-like proteins, and appears to prevent platelet adhesion via a mechanism independent of its enzyme inhibitory activity (Prevot, 2006). However, even if Iris inhibits several serine proteases in coagulation pathways, it does not appear to be a powerful anticoagulant (Prevot, 2006).

**Madanin 1 and 2** have also been identified from SGs of *H. longicornis*, as inhibiting both the intrinsic and extrinsic coagulation pathways (Iwanaga et al., 2003). Madanin proteins do not exhibit any sequence similarities with any other previously identified proteins, do harbor a signal peptide sequence, and interact with thrombin, but not with factor Xa (Iwanaga et al., 2003). Thrombin inhibition by madanins probably involves competitive binding to thrombin's fibrinogen-binding site (anion-binding exosite 1), and not via binding to the active site. They inhibit blood coagulation at an early stage by inhibiting thrombin activation of factors V and VIII, and thus likely contribute considerably to tick blood feeding success (Iwanaga et al., 2003). Another thrombin inhibitor named **Chimadanin** was identified in *H. longicornis* SGs with very weak expression. It was also detected in the hemolymph during nymphal and adult stages from the third day of blood feeding and declines until extinction on the 6th day (Nakajima et al., 2006). Lastly, **Hemalin** with two Kunitz domains, was identified in larval, nymphal, and adult stages, and exhibited highest expression levels during the rapid blood meal sucking period during all tick life stages (Liao et al., 2009). In addition to its role as a thrombin inhibitor, Hemalin also inhibits trypsin activity. Initially discovered in the midgut, Hemalin is also expressed in major tissues of the female tick including SGs, ovaries, hemolymph, and body fat.

In 2014, Ibelli et al. reported the presence of a new tSPI from *I. scapularis* saliva belonging to the serpin family, **IxcS-1E1** (Ibelli et al., 2014). IxcS-1E1 transcript was detected both in SGs and in the midgut of ticks with a dichotomous temporal expression pattern. In SGs, expression is up-regulated from the 24 first hours of feeding, while midgut expression was down-regulated in response to feeding activity (Ibelli et al., 2014). During feeding, IxcS-1E1 is injected into the host and likely prevents platelet aggregation, as it extends clotting time both in aPTT and PT *in vitro* assays. It inhibits thrombin and trypsin activities by forming stable complexes, and also probably inhibits cathepsin G and factor Xa enzymatic activities. Therefore IxcS-1E1 appears to be one of the most important *I. scapularis* saliva proteins mediating tick evasion from the host's hemostatic defense system (Ibelli et al., 2014).

In the *Amblyomma* genus, the **Americanin** protein was isolated from the SGs of *A. americanum*, and was shown to be a slow reversible tight-binding-type thrombin inhibitor (Zhu et al., 1997). Similar to Ixolaris and boophilin, **Amblin** was isolated from the synganglia of engorged *A. hebraeum* females, from where the protein is exported into the hemolymph where it can also be detected (Lai et al., 2004). Without a signal peptide and composed of two Kunitz-like domains, amblin specifically inhibits the thrombin enzyme via an unknown inhibitory mechanism. Crude SG extracts from *A. variegatum*, the tropical bont tick, also exhibited potent anticoagulant activity in the three TT, PT, and aPTT coagulation assays, thus inhibiting at least one factor implicated in the two coagulation pathways (Koh et al., 2007). The TT assay demonstrated the most significant results, indicating that the major targeted factor is thrombin. **Variegin** was then identified as a protein without any similarities to other tSPIs (Kazimírová et al., 2002). Nevertheless, its NH_2_-terminal sequence appears to be a fast competitive tight-binding inhibitor of thrombin. Following HPLC purification, another inhibitor with anti-thrombin effects on human blood platelets and with hirudin-like activity, was also identified from the saliva of *A. variegatum*, but has not been further characterized (Kazimírová et al., 2002).

Hard tick *Hyalomma marginatum rufipes* SG transcriptome investigations unearthed four peptide-encoding sequences, named **hyalomins-1-4**, that showed weak similarity to madanin 1 and 2 from *H. longicornis* (Francischetti et al., 2011). The central core of these polypeptides contains a weakly conserved acidic region that forms a putative serine protease cleavage site. Hyalomin-1 appears to be a specific thrombin inhibitor and noncompetitively binds to its active site, as well as an exosite I, thus significantly extending fibrin clot formation time in whole plasma (Jablonka et al., 2015). It also blocks thrombin activation of coagulation factor XI and factor V. In addition, hyalomin-1 also impedes platelet aggregation by inhibiting thrombin activation of platelet proteinase activated receptor (PAR).

Two thrombin inhibitors from *H. dromedarii* nymphs were also described and named **NTI-1** and **NTI-2** (Ibrahim et al., 2001a). Inhibition assays revealed that NTI-1 inhibits 13% of FXa activity and 65% of thrombin activity, whereas NTI-2 inhibits 100% of FXa activity and 58% of thrombin activity, but with higher affinity for this enzyme than NTI-1. In addition, thrombin inhibition by NTI-1 and NTI-2 is non-competitive and competitive, respectively.

**Rhipilin-1**, identified in the hard tick *Rhipicephalus haemaphysaloides*, shares a similar conformation—a unique Kunitz domain—with other thrombin inhibitors such as boophillin, amblin, and hemalin (Gao et al., 2011). The protein, tested on rabbit citrated plasma, has the capacity to extend both RCT and aPTT in a dose-dependent manner. Both tick attachment and engorgement processes require *rhipilin-1* expression, which is consistent with specific gene expression only in fed ticks (Gao et al., 2011). Two other thrombin inhibiting serpins were also identified in this tick species, **serpin-1** (**RHS-1**) and **serpin-2** (**RHS-2**) (Yu et al., 2013). They share similarities with other tSPIs described in this review including: RHS-1 with lopsins 1 and 2; and RHS-2 with lopsin 7 and RAS-2. Functional characterization indicated that RHS-1 is expressed in SGs of fed ticks and secreted at the tick-host interface during feeding, whereas RHS-2 is only expressed in fed tick midgut (Yu et al., 2013). Inhibition assays showed that both RHS-1 and RHS-2 mostly inhibit chymotrypsin but they also have thrombin inhibitory activity, with maximum inhibition rates of 65.5 and 20%, respectively (Yu et al., 2013). For both, weak FXa inhibition was observed, and neither trypsin nor elastase was inhibited. Anticoagulation assays revealed that aPTT was prolonged in the presence of RHS-1 but not RHS-2. RNAi assays implicated both proteins in tick attachment and engorgement rates, but not in repletion time or average body weight, confirming the role of RHS-1 and RHS-2 in tick blood feeding by blocking blood coagulation (Yu et al., 2013).

**Calcaratin** was identified from *Rhipicephalus* (*Boophilus) calcaratus* and was able to delay coagulation time in all tests (aPTT, TT, and FCT), via an unknown thrombin inhibition mechanism (Motoyashiki et al., 2003).

Concerning soft ticks, ornithodorin was described from *O. moubata* as a thrombin inhibitor harboring two Kunitz domains (van de Locht et al., 1996). Interactions with thrombin implicate its active and exosite: the NH_2_-terminal domain appears to be responsible for the majority of thrombin interaction with two van-der-Waals contacts and hydrogen bonds, whereas the COOH-terminal domain has mostly electrostatic interactions with thrombin (Klingler and Friedrich, 1997). An ornithodorin ortholog was identified in *O. savignyi* SGs, named savignin (Mans et al., 2002). Its NH_2_-terminal region seems to be implicated in binding to the thrombin active site, whereas the COOH-terminal domain helix binds to the fibrinogen-recognition exosite domain. Savignin was then described as a slow competitive tight-binding inhibitor that binds to thrombin's fibrinogen-binding exosite for inhibition. It carries a signal peptide substantiating likely secretion during blood feeding (Mans et al., 2002). Lastly, the **monobin**, a slow specific tight-binding thrombin inhibitor, and an ornithodorin and savignin ortholog belonging to the Kunitz family, was also identified in *Argas monolakensis* (Mans et al., 2008). These three tSPIs harbor a non-canonical mechanism of action as inhibition results when their NH_2_-terminal residues are inserted into the enzyme active site instead of their active Kunitz loops (van de Locht et al., 1996).

Lastly, studies on salivary gland extracts from *Ixodes holocyclus*, the Australian paralysis tick, showed that thrombin was the main enzyme targeted by salivary anticoagulant molecules, with an uncharacterized mechanism (Anastopoulos et al., 1991).

### Vertebrate host-immune modulation by tick serine protease inhibitors

Because ticks are blood-feeding arthropods requiring hours to weeks to complete their blood meal, they have developed several mechanisms to evade host rejection during this long feeding period (Ribeiro, 1995; Francischetti et al., 2009; Kazimirova and Stibraniova, 2013; Kotal et al., 2015). Their saliva injected into the wound site carries many components able to manipulate vertebrate host responses, including anti-inflammatory compounds as well as immunomodulators acting on both innate and acquired immunity, and among which are included several tSPIs.

In *A. americanum*, the aforementioned anticoagulants **AamS6** and **AAS19** can also target plasmin, known for its role in pro-inflammatory cytokine release, monocyte and dendritic cell chemotaxis, neutrophil attraction, tissue remodeling, and wound healing, suggesting involvement in the regulation of monocyte, macrophage, and dendritic cell functions, and in inflammatory responses (Syrovets et al., 2012). In addition, 17 different serpins called **lopsins** (L1-L17) were found to be expressed in various organs such as SGs, midguts, ovaries, and the carcasses of partially fed *A. americanum* ticks (Mulenga et al., 2007). Sequence analysis revealed the presence of glycosaminoglycan binding sites on all lopsins, similar to several other proteins involved in the modulation of blood coagulation, inflammatory responses, or immune cell migration (Munoz and Linhardt, 2004). However, further investigations are required to identify their inhibitory targets and to decrypt mechanisms governing lopsins' likely immunomodulatory activities.

**Iris** from *I. ricinus* is also a powerful immunosuppressive molecule (Leboulle et al., 2002a). It is secreted at the tick-bite site and strongly inhibits elastase-like proteases (leukocyte elastase and pancreatic elastase) with rapid kinetics, thus repressing host inflammation (Prevot, 2006). Iris regulates innate immune mechanisms by suppressing T lymphocyte proliferation and inducing Th2-type immune responses with increased IL-4 and by inhibiting typical Th1 molecule production (IL2, IFN-γ) (Leboulle et al., 2002a). **IRS-2**, another serpin exhibiting specific anti-chymotrypsin activity, was identified from *I. ricinus* SGs (Chmelar et al., 2011). In SGs, IRS-2 is highly expressed at 2 days with maximum expression 6 days after attachment, suggesting a role in the early stage of feeding. IRS-2 inhibits both tissue swelling and neutrophil migration into inflamed tissue, modulates T cell differentiation, and decreases IL-6 production at both protein and mRNA levels in spleen dendritic cells activated by *B. burgdorferi* (Chmelar et al., 2011). In addition, by decreasing STAT-3 signaling molecule phosphorylation, IRS-2 impairs Th17 cell development (Páleníková et al., 2015). IRS-2 is also the only known serpin that targets both cathepsin G and chymase. Both of these proteases are secreted following neutrophil (cathepsin G) and mast cell (chymase) activation, and are involved in a huge range of physiological processes associated with the acute inflammatory response, particularly in crosstalk between neutrophils and platelets (Zarbock et al., 2007). IRS-2 also affects thrombin-induced platelet aggregation, thus likely playing multiple roles in inflammation and hemostasis particularly through the modulation of PAR activation (Chmelar et al., 2011).

The serpin **Ipis-1**, which shares 95.5% sequence identity with Iris, was isolated from SGs of fed *Ixodes persulcatus* and may be associated with immunomodulatory effects on both innate and acquired immune responses (Toyomane et al., 2016). Ipis-1 may directly interact with and inhibit T cells and CD14^+^ cells (mainly macrophages, neutrophils, and dendritic cells), with an as yet unidentified inhibitory mechanism. Similarly, Ipis-1 affects cytokine and chemokine activity via currently unknown mechanisms (Toyomane et al., 2016).

Based on previous *I. scapularis* sialome exploration (Ribeiro et al., 2006), a salivary Kunitz inhibitor with unusual structure was characterized and named **tryptogalinin** (Dai et al., 2012; Valdés et al., 2013). Tryptogalinin has a broad spectrum (potentially explained by the presence of three intrinsic disordered regions) and a high affinity for serine proteases playing an important role in inflammation and host immune responses (Heutinck et al., 2010). In fact, tryptogalinin inhibits trypsin, α-chymotrypsin, plasmin, matriptase, elastase, and especially human skin β tryptase found in mast cells (Valdés et al., 2013). Mast cell β-tryptase has a key role in host-inflammatory responses by stimulating chemoattractant release, such as IL-8, and by inducing IL-1β mRNA expression (Caughey, 2007). Tryptogalinin causes excitation of sensory neurons and has anti-inflammatory activity by repressing tryptase-PAR type 2 activation which normally mobilizes intracellular calcium stores to increase intracellular Ca^2+^ concentrations (Payne and Kam, 2004).

The glycosylated Kunitz inhibitor, **TdPI** (tick-derived protease inhibitor), was isolated from SGs of *R. appendiculatus* adult females (Paesen et al., 2007). *TdPI* is only expressed during the four first hours of feeding and manipulates host-immune defenses during the tick feeding process. TdPI potently inhibits trypsin and moderately affects human plasmin and human tryptase activity (Paesen et al., 2007). Mast cells and eosinophils initiate and/or amplify inflammation at the tick feeding site, mostly via the production and degranulation of several pro-inflammatory molecules such as histamine and tryptase. Accordingly, TdPI transcription coincides with that of RaHBPs (*R. appendiculatus* Histamine-Binding Proteins) known to sequester histamine in ticks (Paesen et al., 1999), thus highlighting a probable role for TdPI as a human tryptase inhibitor via complex formation (Paesen et al., 2007). In addition, TdPI may bind to tryptase inside mast cells, and may suppress its autocatalytic activation step (Sakai et al., 1996).

In *R. (B.) microplus*, **BmSI 7** inhibitor regulates bovine neutrophil elastase pro-inflammation activity, due to its strong elastase inhibitory activity. Thus by reducing inflammation and/or avoiding tick tissue degradation, it enables the tick to thwart the host immune system (Sasaki et al., 2008). Similarly, **RMS-3** is likely secreted into tick saliva during feeding, and carries a B-Lymphocyte epitope, highlighting a probable role in host-immune response modulation (Rodriguez-Valle et al., 2012). Finally, 12 further proteins have been extracted from *R. (B.) microplus* larvae, initially **BmTI A-F**, then **BmTI 1-7**, among which only **BmTI 2** and **BmTI 3** were further characterized as follows (Sasaki et al., 2004). Both BmTIs were classified into the BPTI-Kunitz family as they are suspected to have two Kunitz domains. Both inhibit trypsin and hNE, BmTI 2 demonstrating greater trypsin inhibition, and BmTI 3 stronger hNE inhibition, suggesting a role inhibiting the host's inflammatory response (Sasaki et al., 2004).

### Host angiogenesis and apoptosis induction

While ticks are biting, they must control and limit wound neovascularization and cell proliferation responses to enable blood meal uptake. Thus, they inject salivary proteins to favor apoptosis and slow down neovascularization as reported for *I. scapulari*s (Francischetti et al., 2005a), and interestingly, tSPIs have also been implicated in this role.

Significant similarities are shared between **BmCI** from *R.(B.) microplus* (Lima et al., 2010) and dendrotoxins. Dendrotoxins are non-inhibitory Kunitz proteins able to block different ion channels (Na^+^, K^+^, Ca^+^) involved in both cell proliferation (Lang et al., 2005) and apoptosis (Nutt et al., 2002). BmCI appears to be highly cytotoxic and causes fibroblast cell death via pro-apoptotic activity, without affecting cell cycle integrity, likely by Ca^+^ channel activity regulation (Lima et al., 2010).

**Haemangin** was identified as a salivary Kunitz inhibitor from *H. lonigicornis* carrying one single Kunitz domain, which is up-regulated during blood feeding (Islam et al., 2009). It strongly inhibits trypsin and chymotrypsin, poorly inhibits elastase, and is able to efficiently stimulate degradation of both heavy and light plasmin chains during fibrinolysis, therefore strongly supporting plasmin-dependent fibrinolysis inhibition (Islam et al., 2009). Haemangin also inhibits chick aortic explant angiogenesis; human umbilical vein endothelial cell (HUVEC) differentiation, proliferation, and tube formation; and chick ChorioAlantoic Membrane (CAMs) neovascularization, demonstrating that it can impede with both pre-existing vessel angiogenesis and neovascularization (Islam et al., 2009). Haemangin also significantly induces apoptosis in HUVECs (Nagata, 2000), and affects wound healing in an artificially wounded HUVEC monolayer (Islam et al., 2009). Anti-haemangin RNAi experiments showed that ticks completely failed to make blood pools by 72 h and achieved significantly diminished engorgement by 144 h, while control ticks become engorged by 120 h, with the simultaneous increase of neovascularization in knockdown ticks (Islam et al., 2009). High-throughput studies also indicated that Haemangin can utilize multiple intracellular signaling pathways to negatively regulate angiogenesis, and angiogenesis-dependent wound healing (Islam et al., 2009). Interestingly, it was noticed that soft ticks—which are fast blood-feeders compared to hard tick—lack Haemangin homologs, thereby highlighting the importance of these molecules during the long blood feeding processes of hard ticks (Francischetti et al., 2005a).

**BmTI-A** from *R. (B.) microplus* also strongly inhibits neovascularization, and prevents new vessel formation *in vitro* by inhibiting plasma kallikrein, plasmin, and elastase (Soares et al., 2016). In addition, BmTI-A inhibits endothelial cell viability and proliferation through kallikrein inhibition (Lang et al., 2005), leading to an absence of bradykinin release, which normally stimulates cell growth and survival (Andoh et al., 2010). The inhibition of plasma kallikrein and plasmin by BmTI-A prevents angiogenic growth factor release during tick infestation, thereby averting cell adhesion mechanisms (Soares et al., 2016). Neutrophil elastase degrades a broad spectrum of extracellular matrix and cell surface proteins, and the release of growth factors such as TGF-β, PDGF, and VEGF, induces cell proliferation and migration. By inhibiting neutrophil elastase, BmTI-A could also inhibit angiogenesis (Wada et al., 2007). BmTI-A is also believed to have an inhibitory action on both vessel formation and wound healing, thus enabling *R. (B.) microplus* to continue feeding (Soares et al., 2012).

Finally, the anticoagulant **Amblyomin-X** from *A. cajennense* can also act as a proteasome inhibitor (Chudzinski-Tavassi et al., 2010). Amblyomin-X demonstrates cytotoxicity in different tumor cells lines, but not in healthy human fibroblast cells (Chudzinski-Tavassi et al., 2010; Morais et al., 2016). In human tumor cells, Amblyomin-X alters the expression of several genes related to the cell cycle (related to the G0/G1 phase, as supported by observed G0/G1 alterations), causes endoplasmic reticulum stress marker accumulation, slightly modulates intracellular calcium concentration [Ca2^+^], causes mitochondrial dysfunction, cytochrome C release, poly (ADP-ribose) polymerase (PARP) cleavage, and activates the caspase cascade (Chudzinski-Tavassi et al., 2010; Morais et al., 2016). Additional *in vitro* assays on endothelial cells showed that Amblyomin-X delays the cell cycle, inhibits cell proliferation and adhesion, tube formation, and membrane expression of the platelet-endothelial cell adhesion molecule-1 (PECAM-1) (Drewes et al., 2012). *In vivo*, Amblyomin-X reduces tumoral mass in a murine melanoma model, decreases the number of metastatic events (Chudzinski-Tavassi et al., 2010), and also inhibits VEGF-A-induced angiogenesis in the dorsal subcutaneous tissue in mice (Drewes et al., 2012). Similar effects were observed in chicken chorioallantoic membrane (CAM). These investigations confirm the anti-tumorigenic and anti-angiogenic properties of Amblyomin-X.

## Serine protease inhibitors involved in tick-borne pathogen development and transmission

During tick feeding, all the above-mentioned molecules modulating host immune responses and homeostasis create an environment conducive to pathogen transmission and host infection (Brossard and Wikel, 2004; Nuttall and Labuda, 2004; Ramamoorthi et al., 2005; Wikel, 2013). Several tick molecules directly implicated in pathogen transmission have also been identified. They either facilitate pathogen development in the tick vector, or enhance pathogen transmission to the vertebrate host (Liu and Bonnet, 2014). Among them, a few tSPIs have been shown to affect pathogen development and/or transmission (Figure 2, Table 2) but, in all cases, the precise mechanisms involved remain unknown.

Two-dimensional electrophoresis of total proteins from fully engorged *R. (B.) microplus* adults revealed **BmTI-A** up-regulation in *B. bovis*-infected ticks. This differential expression suggests a putative role for this protein in the tick's immune system, to either limit the potentially detrimental proliferation of the parasite in the vector, or as a molecule required for parasite development and/or colonization of tick tissues (Rachinsky et al., 2007). It is noticeable that the expression of BmTI-A in tick ovaries is coherent with the vertically transmission that occurs for this parasite from the female to the next generation.

**DvKPI** from *D. variabilis* reduces burden of the obligate intracellular bacteria *R. montanensis* at 24 h post-infection in L929 mouse fibroblasts *in vitro* (Ceraul et al., 2008), and associations between the inhibitor and the bacteria have been reported, thus it was hypothesized that bacteria may express trypsin-like proteases on their outer surface (Ceraul et al., 2011). Alternatively, DvKPI may be implicated in recruiting host factors, such as plasminogen activation system factors facilitating midgut epithelium transmigration, as has been demonstrated for *B. burgdorferi* (Coleman et al., 1997). Therefore DvKPI might be involved in both the tick immune system and in *Rickettsia* development (Walker et al., 1984).

In *I. ricinus*, a comparison of *Bartonella henselae*-infected or non-infected SG transcriptomes led to the discovery of **IrSPI** (*Ixodes ricinus* serine protease inhibitor), a Kunitz inhibitor with the highest expression following bacterial infection (Liu et al., 2014). It was demonstrated that *B. henselae*, a facultative intracellular bacterium responsible for cat-scratch disease, can be transmitted by *I. ricinus* through the injection of saliva (Cotté et al., 2008). RNAi experiments showed that when *IrSPI* expression was significantly knocked down, both the weight of engorged ticks and *B. henselae* SG load were significantly decreased, suggesting IrSPI involvement in blood feeding, as well as in SG bacterial development (Liu et al., 2014).

Lastly, Lyme disease caused by bacteria from the *Borrelia* genus is unquestionably the predominant concern for the Northern latitude (Dantas-Torres et al., 2012) and several studies concern their transmission by tick from the *Ixodes* genus. **Ixophilin**-immunized mouse assays showed that despite no effects on *Borrelia burgdorferi* burden in *I. scapularis* tick midgut, significantly increased *B. burgdorferi* burden in the skin, heart, and bladder of immunized mice was observed, suggesting a probable role in *B. burgdorferi* transmission to and/or development in the host (Narasimhan et al., 2013).

## Applications in tick control

To date, acaricides are mainly used to control ticks. But their use has several detrimental effects, including a negative impact on the environment and non-targeted species, as well as an associated rise in resistant tick strains. Thus new effective control measures are urgently required, such as anti-tick vaccines. Vaccines that target important molecules implicated in tick feeding processes and physiology could decrease tick populations and limit transmission of a vast number of pathogens. This is the manner in which the only two commercially-available anti-tick vaccines—based on an *R. (B.) microplus* midgut protein—are believed to function (Kemp et al., 1989). By targeting tick molecules implicated in pathogen establishment, development and transmission, vaccines could also completely impair pathogen transmission.

Several tSPIs have been investigated as possible targets to create anti-tick vaccines. First, a combination of **RAS-1 and -2** *R. appendiculatus* serpins was tested on cattle (Imamura et al., 2006). Results showed that the cumulative number of engorged nymphs and adults fed on vaccinated cattle was significantly lower compared to controls, tick mortality rates were significantly higher, and eggs masses from females were lower. But for ticks that did feed, feeding duration as well as engorgement weight did not differ between the two tick groups.

**BmTIs** from *R. (B.) microplus* have also been used in cattle immunization assays. A reduction in the total number of engorged ticks was observed (72.8%), as well as reduced engorged female weight in vaccinated animals comparing to controls (Andreotti et al., 2002).

The Serpin-1 **(HLS-1)** from *H. longicorni*s was also evaluated as a vaccine candidate against tick infestation (Sugino et al., 2003). No differences in feeding duration or engorgement weight between vaccinated rabbits and controls groups were observed, but a significant increase in tick mortality rates was reported for ticks fed on vaccinated animals. As rabbit anti-tick immunity compromised tick physiology of both nymphs and adults, these promising results support HLS-1 as a vaccine cocktail component, along with other previously characterized antigens (Mulenga et al., 1999; Tsuda et al., 2001).

Another trypsin inhibitor from *R. (B). microplus* was recently identified due to its homology with BmTI. Named **RmLTI**—for *R. (B.) microplus* larval trypsin inhibitor—it belongs to the Kunitz inhibitor family (Guerrero et al., 2005; Andreotti et al., 2012). RmLTI's role is completely unknown, but due to encouraging results reported for BmTIs, RmLTI's potential as a vaccine antigen was also tested in cattle. Vaccination with recombinant RmLTI showed that the average weight of engorged ticks was significantly lower after feeding on vaccinated cattle for up to 9 days after vaccination, but then remained equivalent from days 9–13, probably due to declining antibody levels (Andreotti et al., 2012). However, ticks detaching from RmLTI-immunized cattle still appeared to be affected at day 13, and while no differences were observed in egg laying, significant effects were observed on egg viability, eclosion rate, as well as larval hatchability (Andreotti et al., 2012).

## Conclusion

The goal of this review was to comprehensively describe the varied roles of tSPIs in both tick physiology and vertebrate host response modulation following tick bite, emphasizing their vital roles in tick-host-pathogen interactions. tSPIs are thus involved in essential processes such as tick's innate immune system and hemolymph clotting. In addition, some tSPIs expressed in ovaries and eggs are essential for tick development, oviposition and egg laying. Some ovary-expressed inhibitors are also utilized by eggs to avoid self-proteolysis and protection against foreign micro-organisms. Ticks have been obligatory blood feeding arthropods for more than 90 million years, as such, they've developed and adapted appropriate molecules to facilitate their extremely efficient feeding processes. Several tSPIs are implicated in blood uptake and digestion in the tick midgut, SGs, and at the wound feeding pool on the host. In addition to immunomodulation and angiogenesis suppression, most tSPIs inhibit blood coagulation, often via FXa and thrombin targeting, enabling effective blood intake. Some tSPIs have also been reported to be involved in direct pathogen establishment and/or transmission, and also by creating opportune conditions to facilitate pathogen transmission from ticks to hosts. Such a wide spectrum of actions ensures that tSPIs are attractive and promising target candidates in anti-tick vaccine strategies to block tick feeding and/or TBP transmission.

## Author contributions

AB and SB conducted the literature research and prepared the figures and tables. SB, AB, and TF wrote the paper, provided critical review, and revisions.

### Conflict of interest statement

The authors declare that the research was conducted in the absence of any commercial or financial relationships that could be construed as a potential conflict of interest.

## Abbreviations

α2M: alpha 2-macroglobulin
APC: antigen presenting cells
aPTT: activated partial thromboplastin time
BPTI: bovine pancreatic trypsin inhibitor
BSAP: BaSO4^−^ adsorbing protein
CAMs: chorioalantoic membranes
Efh: EF hand
FCT: fibrinogen clotting time
FRP: follistatin-related-protein
HBE: heparin binding exosite
HMWK: high molecular weight kininogen
hNE: human neutrophil elastase
hTFPI: human tissue factor pathway inhibitor
HuPK: human prekalikrein
HUVEC: human umbilical vein endothelial cells
LICI: limulus intracellular coagulation inhibitor
MA: methyl amine
PAR: proteinase activated receptor
PARP: poly (ADP-r-ribose) polymerase
PDGF: platelet-derived growth factor
PECAM-1: platelet-endothelial cell adhesion molecule-1
PPE: porcine pancreatic elastase
PT: prothrombin time
RCL: reactive center loop
RCT: recalcification time assay
RNAi: RNA interference
SGs: salivary glands
STAT 3: signal transducer and activator of transcription 3
STI: soybean trypsin inhibitor
TBP: tick-borne pathogens
TF: tissue factor
TGF-alpha: tissue growth factor alpha
TIL: trypsin inhibitor-like domain
tSPI: tick serine protease inhibitor
TT: thrombin clotting time
VEGF: vascular endothelial growth factor.
